# Effect of Protonation on the Molecular Structure of Adenosine 5′-Triphosphate: A Combined Theoretical and Near Edge X-ray Absorption Fine Structure Study

**DOI:** 10.1021/acs.jpclett.3c01666

**Published:** 2023-11-05

**Authors:** Giuseppe Mattioli, Robin Schürmann, Chiara Nicolafrancesco, Alexandre Giuliani, Aleksandar R. Milosavljević

**Affiliations:** ‡ Istituto di Struttura della Materia (ISM), Consiglio Nazionale delle Ricerche (CNR), Area della Ricerca di Roma 1, CP 10, 00016 Monterotondo Scalo, Italy; § Synchrotron SOLEIL, L’Orme de Merisiers, Saint Aubin, BP 48, 91192 Gif-sur-Yvette Cedex, France; ∥ Institute of Chemistry, University of Potsdam, 14476 Potsdam, Germany; # Normandie Université, ENSICAEN, UNICAEN, CEA, CNRS, CIMAP, 14000 Caen, France; ∇ INRAE, UAR1008, Transform Department, Rue de la Géraudière, BP 71627, 44316 Nantes, France

## Abstract

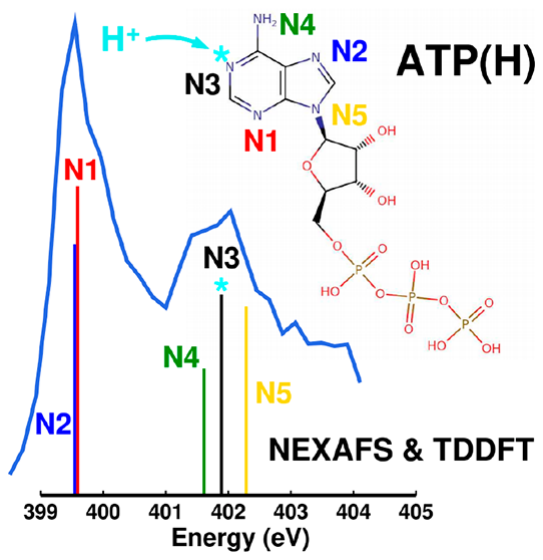

The present work combines the near edge X-ray absorption mass spectrometry of a protonated adenosine 5′-triphosphate (ATP) molecule isolated in an ion trap with (time-dependent) density functional theory calculations. Our study unravels the effect of protonation on the ATP structure and its spectral properties, providing structure–property relationships at atomistic resolution for protonated ATP (ATPH) isolated in the gas-phase conditions. On the other hand, the present C and N K-edge X-ray absorption spectra of isolated ATPH appear closely like those previously reported for solvated ATP at low pH. Therefore, the present work should be relevant for further investigation and modeling of structure–function properties of protonated adenine and ATP in complex biological environments.

Adenosine 5′-triphosphate (ATP) is an extraordinary molecule that acts as one of the main energy containers and transporters in living organisms, capable of storing and releasing energy for high-level, complex functions inside a cell but also serving as a critical signaling molecule between the cells.^1^ To accomplish this challenging role, a complex chemical structure is needed through the connection of three units having different functions. Such units contain either different atomic species (N is present only in adenine, and P is present only in phosphates) or the same atomic species but have significantly different local environments (conjugated C in adenine is quite different from saturated C in ribose, and C–O bonds are quite different from P–O bonds). For such reasons, ATP has been attracting significant scientific attention since its discovery in 1929.^1,2^ Gas-phase spectroscopy represents an invaluable tool to probe the electronic properties of isolated, well-defined molecular systems, which can be directly interpreted with the help of atomistic simulations to unravel structure–property relationships. Development of experimental techniques, such as electrospray ionization (ESI),^3^ allowed for spectroscopic studies of isolated ATP or adenosine monophosphate (AMP) ions, such as ultraviolet (UV) laser (4.0–5.8 eV) photodissociation,^4^ anion photoelectron imaging,^5^ and photoelectron spectroscopy (PES)^6^ of gas-phase deprotonated ATP/AMP molecules as well as comprehensive collisional and electron-based tandem mass spectrometric studies.^7−9^ Near-edge X-ray absorption fine structure (NEXAFS) and X-ray photoelectron spectroscopy (XPS) have not been reported for isolated ATP (neutral or ionic) and are limited to isolated ATP subunits.^10^ Core-level spectroscopies, however, offer unique possibilities to probe the electronic properties of a desired system and are particularly sensitive to (de)protonation and environmental effects. Moreover, protonation correlated with core-level spectroscopies represent a useful tool to probe chemical properties, such as rates of electrophilic substitutions,^11,12^ promoting an in-depth understanding of molecular reactivity in complex biological environments.

Studies on isolated ATP cannot be a true analogue for real biological systems but could provide relevant insights. For example, Frańska et al. recently investigated gas-phase decompositions of electrosprayed magnesium complexes with ATP and adenosine 5′-diphosphate (ADP) in the negative ion mode and concluded that the presence of the phosphate–metal–nucleobase interaction may also be important for biological processes.^13^ Development of the liquid microjet experimental technique allowed for performance of the first NEXAFS study of solvated ATP.^14,15^ Shimada et al^15^ probed the nitrogen K-edge excitation and investigated structural changes of nucleic acid base in aqueous solution by comparing results for AMP and ATP. Kelly et al.^14^ probed the carbon and nitrogen K-edges for different pH and metal complements and pointed out that such studies would lead to more reliable models of ATP bound inside the enzymes. Besides the interest in many enzymatic reactions involving ATP,^16^ there has been intensive research to understand the signaling role of ATP and how it binds to dedicated cell receptors; the first crystal structure of such a receptor has been revealed only about 10 years ago.^17^ While the latter liquid-jet studies report unprecedented information on solvated ATP (ATP_aq_), they still leave open questions. For example, they do not show how the resonant core-level photoexcitation (and the underlying structural properties) changes from isolated adenine to neutral ATP and then to protonated ATP ions for different protonation sites. Moreover, they are not accompanied by first-principles calculations to discuss possible ATP structure–property relationships. The present study combines NEXAFS at the C, N, and O K-edges of isolated, singly protonated ATP molecules (ATPH^+^) along with density functional theory (DFT)/time-dependent density functional theory (TDDFT) calculations to resolve the effect of protonation on the ATP structure and its spectral properties, thus including and comparing in the same study results arising from gas-phase and solvated ATP. Although ATP is anionic in solution under neutral pH, the protonation of the adenine unit has been suggested to occur in solution, thereby leading to a sort of salt-bridged structure.^18,19^ The protonation of adenine and possibly its interaction with negatively charged phosphate affect the structure of various types of RNA.^20,21^ Previous studies have reported that different protonation patterns produced in solution are, at least partially, preserved in the gas phase upon electrospray ionization.^22^ Hence, a correspondence may be established between gas- and solution-phase studies. Our study of protonated, isolated ESI ions, accompanied by a comprehensive theoretical modeling of neutral and protonated systems, should, therefore, be relevant at least as a starting point for the study of such molecules in solution or even in a biological environment.

Figure 1a sketches the molecular structure of ATP. Nitrogen atoms, present only in the adenine part of the molecule, are labeled 1–5. The atoms N1–N4 are possible protonation sites. Kelly et al.^14^ reported the p*K*_a_ of N3 to be 4.16 and considered it as a single protonation site of ATP. We have performed a comprehensive set of multilevel tight-binding and (time-dependent) density functional theory calculations to closely investigate the structure of protonated ATP and its correspondence to the measured NEXAFS spectra. The calculations include the exploration of rotational isomers of ATP in the gas phase, the exploration of all possible protonated isomers (protomers), DFT calculations of free energies of such four protomers after full geometry optimization, and finally, TDDFT calculations of NEXAFS spectra (see the Experimental and Computational Details and the Supporting Information for more details). For different protonation sites (see Figure 1a), the relative free energies of protomers are listed as follows: protomer 1, +0.05 eV; protomer 2, +0.09 eV; protomer 3, 0.0 eV; and protomer 4, +0.53 eV. Such values have been calculated using an accurate series of calculations rooted on DFT, as discussed in detail in section S2 of the Supporting Information. Therefore, as reported in a previous study,^14^ the preferred protonation site in the gas phase based on energetics corresponds to N3. The optimized DFT structure of this protomer is presented in Figure 1b, showing a common feature of all protomers in the gas phase, which assumes that coiled structures driven by hydrogen bonds formed between adenine N–H and phosphate P=O moieties (see Figure S9 of the Supporting Information). However, relative energies of protomers 1 and 2 are also not far. Therefore, according to the calculations, those too could be populated in an ion packet of protonated ATP isolated in the gas phase at room temperature. We further performed a detailed combined experimental and theoretical analysis of N and C K-edge spectra of adenine, ATP, and ATPH^+^ to identify the most likely protonation sites. Measured NEXAFS of ATPH^+^ has been performed by coupling a commercial linear ion trap mass spectrometer to a soft X-ray beamline at the SOLEIL synchrotron (see the Experimental and Computational Details for details).

**Figure 1:**
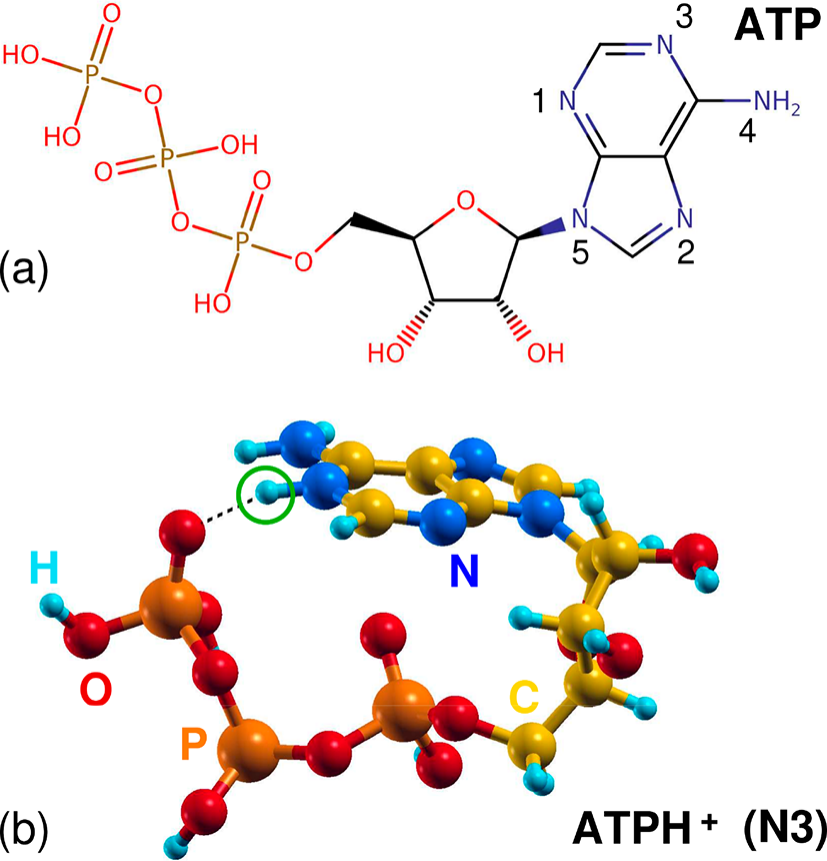
(a) Two-dimensional (2D) structural model of the ATP molecule. Numerical labels 1–5 have been assigned to the five N atoms of the adenine unit to identify protonation patterns. (b) Geometry optimized using the dispersion-corrected M06-2X functional and the def2-TZVPP basis set of one of the investigated ATPH^+^ protomers, protonated at position N3. The additional proton is enclosed in a green circle. A strong hydrogen bond between such a proton and one of the phosphate oxygens, shown as a dashed line, promotes the formation of a coiled molecular structure in the gas phase. ATP and other protomers are also shown in Figure S9 of the Supporting Information.

Figure 2a compares the normalized experimental NEXAFS spectra at the N K-edge of ATPH^+^ (present measurement, light blue plot) with solvated ATP at pH 2.5 (dark green plot) and pH 7.5 (purple plot)^14^ and with gaseous adenine (orange plot).^10^ Note that NEXAFS of solvated ATP acquired for pH 7.5 changes significantly from that at pH 2.5,^14^ with neither closely resemble that of gaseous adenine. The present measured NEXAFS of protonated ATPH^+^ is highly comparable to the solvated ATP at low pH, where most of the ATP molecules are also protonated ATPH^+^ cations, as opposed to pH 7.5, which is compatible with a prevalence of neutral ATP. We consider it a remarkable fact that two significantly different experimental techniques used to study ATPH^+^ yield such closely similar spectra; in the present case (total ion yield within a limited *m*/*z* ratio region; see the Experimental and Computational Details for details), we collect and measure isolated ATPH^+^ ions, while in the case of solvated ATP at pH 2.5, the curve was obtained as the total electron yield of a sample also containing water molecules from the starting solution. Minor discrepancies between the two spectra as a result of differences between the experimental techniques are discussed in detail in section S1 of the Supporting Information. Moreover, in the latter case, water molecules from the solution can interact with ATPH^+^ ions, in principle inducing changes in C and N core-level spectra of biomolecules.^23^ In section S6 of the Supporting Information, we show that the possible presence of water has a minimum effect on the shape and position of N K-edge NEKAFS spectra of ATP and ATPH^+^, justifying direct comparison between the present and previous results.

**Figure 2:**
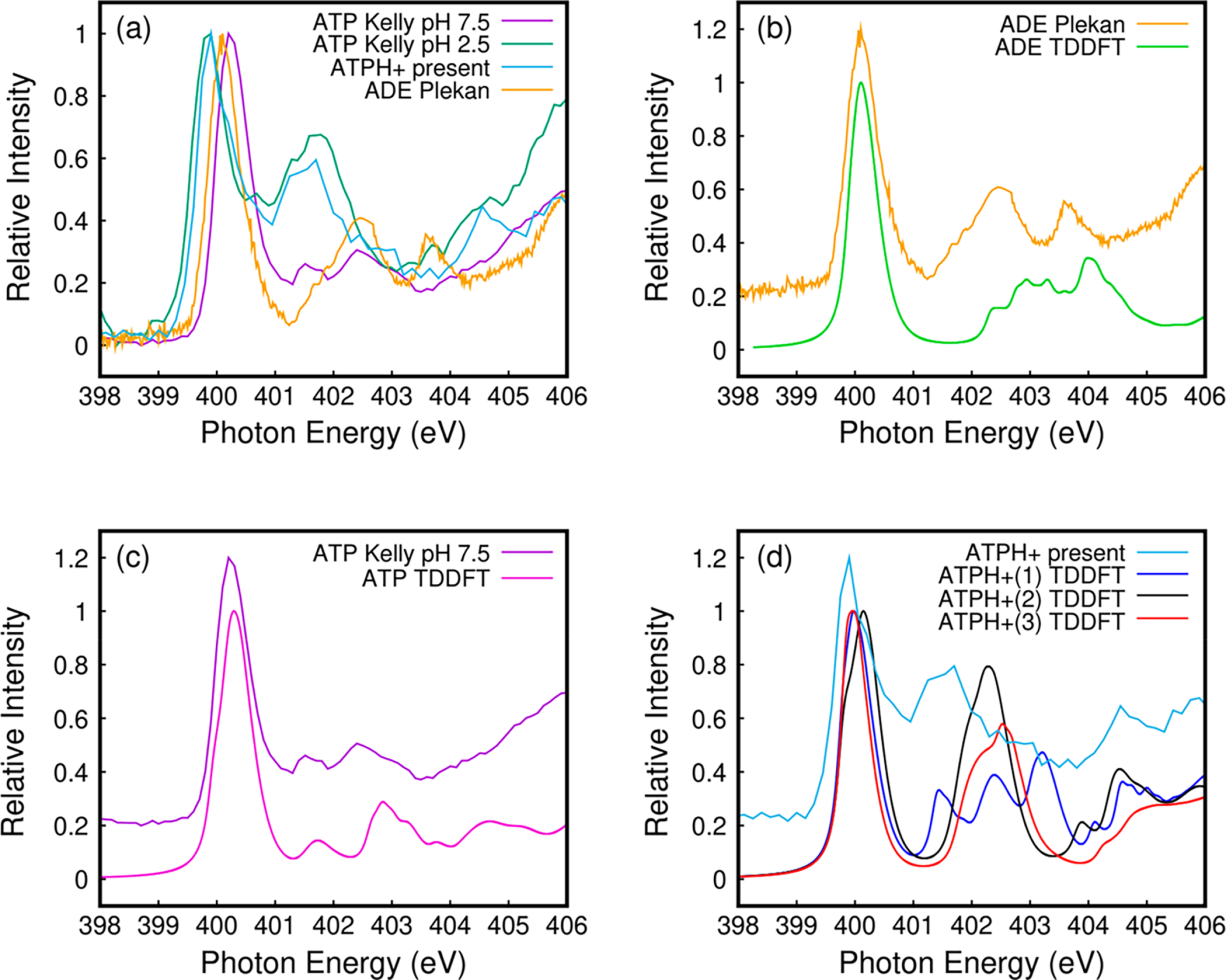
N K-edge NEXAFS. (a) Experimental NEXAFS spectra^24^ of protonated ATP (light blue), solvated ATP at pH 2.5 (dark green) and pH 7.5 (purple),^14^ and gaseous adenine (orange).^10^ Comparison between experimental and theoretical spectra of (b) adenine, (c) ATP, and (d) protonated ATPH^+^. In the last case, 1, 2, and 3 correspond to different protomers discussed in the text.

We note that both ATPH^+^ NEXAFS spectra are markedly different from that of ATP as well as from that of gas-phase adenine. We anticipate here and discuss in detail in section S3 of the Supporting Information that the present calculations, also in agreement with previous theoretical results,^10^ indicate that the first NEXAFS transition arising from N4 (see labels in Figure 1a) is significantly blue-shifted in adenine with respect to ATP and ATPH^+^, opening a gap between the first and second NEXAFS peaks.

Panels b, c, and d of Figure 2 show a comparison between measured and calculated NEXAFS of adenine, ATP, and ATPH^+^, where the three lowest energy protomers introduced above (blue, black, and red plots for protomer 1, protomer 2, and protomer 3, respectively) have been considered. TDDFT calculations show a close match to adenine and ATP measurements. With regard to ATPH^+^, all three spectra reproduce the main differences induced by protonation of ATP, i.e., a moderate red shift of the first, sharp peak of ATPH^+^, a significant increase in the relative intensity of the second, broader band, and the appearance of a further prominent feature around 404.5 eV. In more detail, protomer 1, slightly less stable than protomer 3 by 0.05 eV, is characterized by a broad second band with a width more compatible with measurements. However, protomers 2 and 3 are better suited to reproduce the two-peak structure of the spectrum, even considering a 0.5 eV blue shift of their high-energy contribution. We discuss here the interpretation of NEXAFS changes upon protonation, addressing the reader to section S3 of the Supporting Information for a thorough analysis based on TDDFT results. Protonation reduces the distribution of electronic charge around the core shell of the involved N atom, inducing a ∼2 eV blue shift of the first core-level excitation of the corresponding N atoms. Such a shift changes the balance between the first and second peaks, lowering the former (which, however, remains sharp) and raising the latter, which is also broadened because its three components are more spread. It should also be noted that the second, broad band is systematically red-shifted in ATPH^+^ and ATP with respect to adenine, resulting in a reduction of the gap between the first and second peaks. Such a shift is likely due to the formation of the coiled structures shown in Figure 1 for protomer 3 and in Figure S9 of the Supporting Information for ATP and protomers 1 and 2, driven by the formation of hydrogen bonds between the adenine unit and the phosphate unit. Electron-rich P=O moieties provide a good screening of core holes in the final state of excitations involving, in particular, the −NH_2_ group, pointing outward, thus inducing a significant red shift (0.5–0.7 eV) of the contribution of N4 in ATP and protomers 2 and 3 with respect to adenine. Interestingly, a different mechanism involves protomer 1. As detailed in section S3 of the Supporting Information, in particular, Figure S6 of the Supporting Information, the protonation of N1 induces a peculiar delocalization mechanism that results in a significant enhancement of the sp^2^ character of N4 and a more pronounced (1.0 eV) red shift of its contribution to the spectrum. For this reason, protomer 1 is characterized by a broad distribution of the second block of transitions involving N1, N4, and N5, which is apparently less similar to measurements with respect to protomers 2 and 3.

The present results show that at least two protomers should be considered as representative structures of the protonated ATP molecule, instead of just one typically presumed in the literature. Moreover, the remarkable resemblance between gas-phase and solvated (at low pH) ATP suggests then that such calculated structures could also be relevant as a starting point in modeling the structure of hydrated and solvated ATP, as discussed in more detail in section S6 of the Supporting Information, where the effect of water molecules on structures and spectra is discussed. However, our combined theoretical/experimental study shows that NEXAFS data at the N K-edge alone are thus far not sufficient to unambiguously identify one of the protomers as the only (or most abundant) species. Further analysis of C spectra is therefore required to gain more insight. Indeed, it is not surprising that in a π-conjugated moiety, such as adenine, the protonation and subsequent screening of the localized positive charge affects not only N atoms but also neighboring C atoms. As in the case of N1s, C1s NEXAFS of acidic solvated ATP^14^ and isolated protonated ATPH^+^ match well together and are both quite different from C1s NEXAFS of neutral solvated ATP and isolated adenine^10^ (Figure 3a). However, while in the case of N1s (Figure 2a), NEXAFS of ATP_aq_ at pH 2.5 and isolated ATPH^+^ are very similar; they differ more in the case of C1s. The spectrum at pH 2.5 resolves three features below 288 eV, whereas the spectrum at pH 7.5 resolves only two features (see ref (14)).

**Figure 3:**
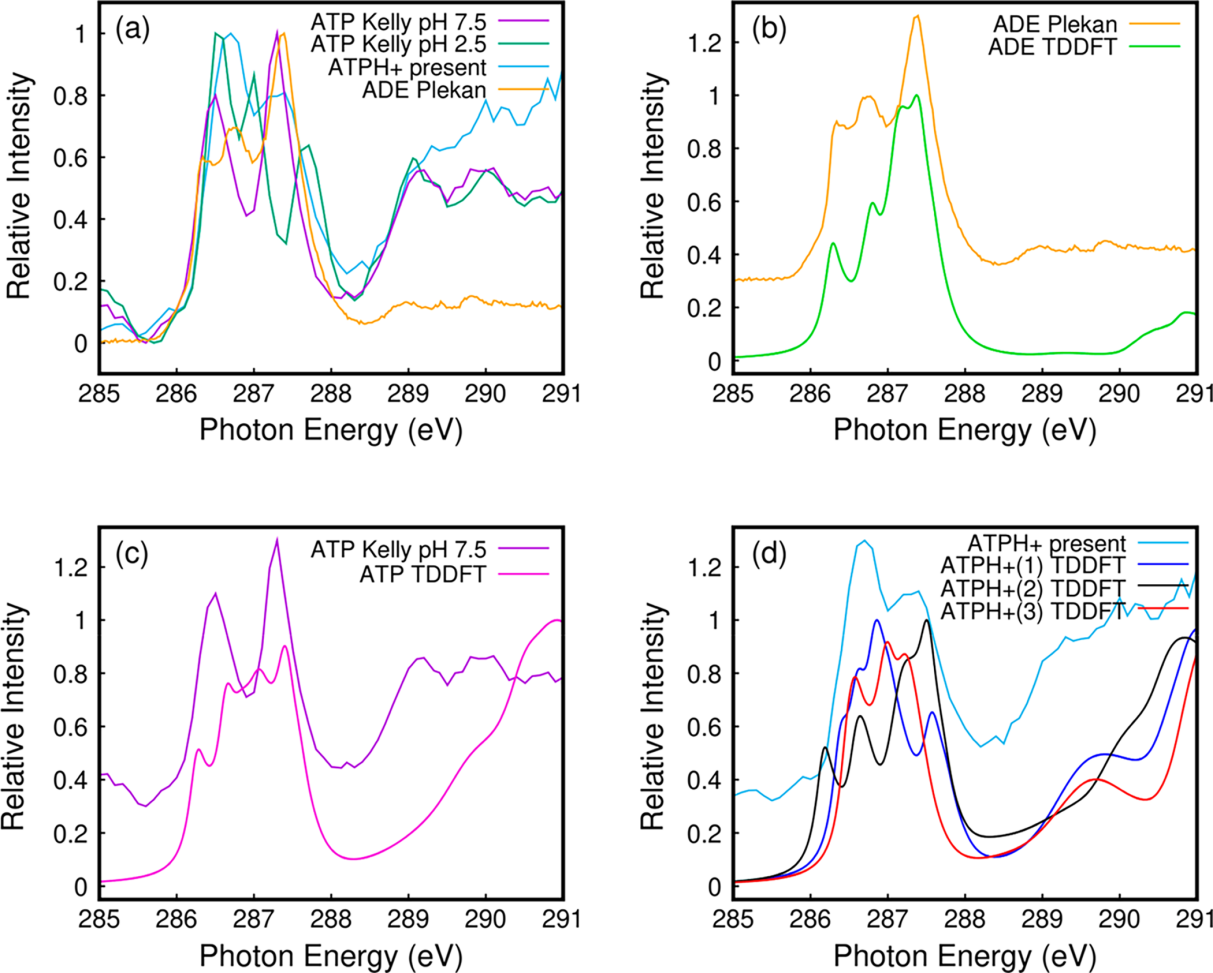
C K-edge NEXAFS. (a) Experimental NEXAFS spectra^24^ of protonated ATP (light blue), solvated ATP at pH 2.5 (dark green) and pH 7.5 (purple),^14^ and gaseous adenine (orange).^10^ Comparison between experimental and theoretical spectra of (b) adenine, (c) ATP, and (d) protonated ATPH^+^. In the last case, 1, 2, and 3 correspond to different protomers discussed in the text.

With regard to TDDFT calculations, there is again a nice matching between simulations and measurements in the case of adenine (Figure 3b) and neutral ATP (Figure 3c) in the 286–288 eV region. The calculated curves for the three protomers are shown in Figure 3d in comparison to those of isolated ATPH^+^, and a more detailed excitation analysis is given in section S3 of the Supporting Information. First of all, only C atoms belonging to the adenine unit are involved in the lowest energy feature of the spectrum, while the presence in the second feature of those belonging to the ribose unit is responsible for the significant difference with respect to isolated adenine in the region between 288 and 291 eV. If we focus on the first peak, we note differences between adenine/ATP, having a similar shape, and ATPH^+^. This is a first indication that protonation of N also affects charge distribution on adenine C atoms. The protomer 2 spectrum is more like ATP and less compatible with ATPH^+^ measurements, because of either its “three-horns” shape with increasing intensity or its red-shifted onset with respect to measurements. On the other hand, both lowest energy protomers 1 and 3 are compatible with experimental NEXAFS.

Figure 4a shows the O1s NEXAFS of ATPH^+^ (light blue curve), which is compared to O1s NEXAFS of thymidine monophosphate (TMP, brown curve) from a thin film^25^ and with TDDFT simulations in Figure 4b. We compare the experimental O1s NEXAFS to that of TMP because, to the best of our knowledge, there are no reported results for ATP in this energy region. The present spectrum is similar to the previously measured O1s spectrum of TMP,^25^ with the exception of the thymine carbonyl band falling at 532 eV, which is not present in adenine. On the other hand, the signal above ca. 534 eV that includes excitation of oxygen atoms in the sugar and phosphate parts is in good agreement with the present data. The present spectrum appears sharper, with two well-distinguished features at 536 and 538 eV, likely because the target is a single molecular ion in the gas phase rather than a molecular film in the condensed phase. The double-resonant feature is well-reproduced by calculations as well. However, the convolution of a large number of weak electronic transitions involving O atoms, whose 1s electrons are excited in orbitals mainly localized on adenine, yields theoretical spectra for protomers 1–3, which are quite similar to each other and also close to neutral ATP. Hence, as expected, oxygen core electrons are not significantly affected by ATP protonation. As a result of similar considerations, we do not show here NEXAFS results measured at the P L-edge, which are of potential interest for a subsequent study of different fragmentation channels following excitation in these molecules but are of no help in the present interpretation of structure–property relationships.

**Figure 4:**
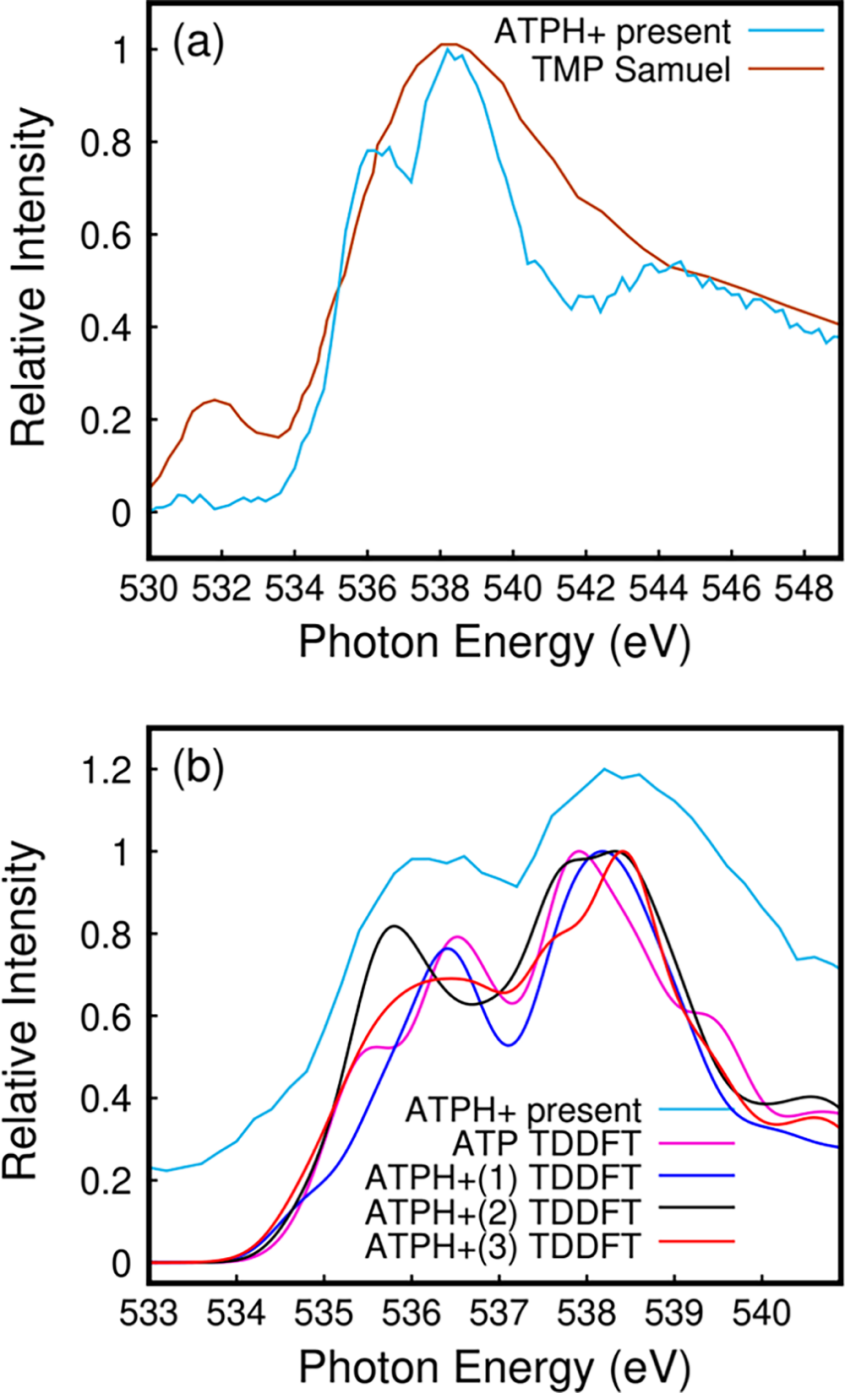
O K-edge NEXAFS. (a) Experimental NEXAFS spectra of protonated ATP (light blue) and a thin film of thymidine monophosphate (brown).^25^ (b) Comparison between the experimental and theoretical spectra of ATP and protonated ATPH^+^. In the latter case, 1, 2, and 3 correspond to different protomers discussed in the text.

In conclusion, we have performed a combined experimental and theoretical study of ATP in the gas phase, using a comprehensive perspective including adenine as a precursor and protonation of the neutral ATP molecule, with the first measurements of protonated ATPH^+^ in the gas phase at the C, N, and O K-edges by NEXAFS. Such an experimental picture of this set of systems has then been enriched and interpreted with the help of DFT/TDDFT simulations, which are, on the one hand, capable of reproducing NEXAFS measurements and, on the other hand, capable of relating electronic properties to well-defined atomistic structures. Our theoretical results also suggest that the most stable folded or coiled structures found in the case of isolated molecules are preserved in gas-phase clusters containing a few water molecules and are stabilized rather then disrupted even in water solution. The comparison between measurements and simulations is striking in the case of adenine and ATP, where tautomerism is excluded. With regard to protonation, the calculations suggest that the three lowest energy protomers 1–3 involving different N atoms of the adenine unit are close in energy in the gas phase, with a slight prevalence of protomer 3, and reveal that, irrespective of the protonation site, predictable and slightly different changes in the inner-shell C and N photoexcitation with respect to neutral ATP can be induced in ATPH^+^ by the addition of one proton. A closer analysis of all of the results suggests that protonation of N3 rather than N1 or N2 yields calculated spectra in slightly better agreement with measurement, also confirming its slight prevalence based on energetics. However, neither differences between the calculated energies nor between the NEXAFS spectra of the protomers are large enough, so that the concurrence of more protomers in the measured sample can be simply ruled out. This finding is important for further modeling of the ATP_aq_ structure, that is, ATP embedded in the water network, toward a better understanding of its messaging function in living organisms.

The present analysis shows that simulations and measurements can be mutually reinforced by closely comparable results. When this happens, it is possible to move spectroscopic studies of complex molecules having biological activity of primary importance a step ahead, even approaching further, more complex atomistic modeling that we just started to explore, capable of accounting for environmental effects, particularly hydration, and finally modeling biological activity through a constant match between theoretical and experimental probes.

## Experimental and Computational Details

*Experiment*. The experimental setup has been described before^26,27^ and is based on a commercial linear ion trap mass spectrometer coupled to a soft X-ray beamline PLEIADES at the synchrotron radiation facility SOLEIL (France). The singly protonated ATP ions are produced by an ESI source from a water/methanol (50:50) solution at 50 μM with 1% formic acid. ATP was purchased from Sigma-Aldrich. The precursor ions are isolated in the linear ion trap and submitted to X-ray radiation during about 400 ms, and then a tandem mass spectrum (MS^2^) is collected after another 50 ms to reduce the background. The MS^2^ spectra were measured between the low mass cutoff at *m*/*z* 115 nm and the precursor mass. Hence, some of the small charged fragments whose mass-to-charge ratio was below 115 may have escaped detection, which may potentially affect the total ion yield. The photon beam is introduced from the back side of the trap. The photon energy was changed in small steps of 0.2 eV, and MS^2^ was acquired during about 10–15 min. For each NEXAFS scan, under the same conditions, the corresponding energy dependence of the photon flux was measured by a photodiode (AXUV-100, IRD) placed in a differentially pumped vacuum chamber upstream from the ion trap. The NEXAFS spectra were obtained as a sum over all MS^2^ fragment ions, normalized to the photon flux and the total ion current (or precursor intensity). The photon radiation is produced by a permanent magnet APPLE II type undulator, with a period of 80 mm, and then monochromatized using a high-flux 600 l mm^–1^ grating and appropriate exit slits. For the present experiment, the energy resolution of the beamline was about 0.5, 0.4, and 0.6 eV for C, N, and O K-edges, respectively. The photon energy was calibrated according to C1s and O1s → π* transition in CO_2_^28^ and N1s(*v* = 0) → π_g_*(*v*′ = 0) transition in N_2_.^29^ The calibration gas was introduced to the calibration chamber (upstream of the ion trap) by an effusive jet crossing at right angles with the synchrotron beam, and the total ion yield was measured using a channeltron. The calibration was performed several times during the experiment before or after each NEXAFS scan. The overall accuracy of the photon energy calibration was estimated to be about 100 meV, not including the error of the literature value for CO_2_ and N_2_.

*Calculations*. Atomistic simulations of all of the investigated molecules have been carried out following a multilevel protocol. Preliminary wide screening of molecular configurations have been performed using a conformer–rotamer ensemble search tool (CREST) rooted on the tight-binding GFN2-xTB Hamiltonian, as implemented in the xTB suite of programs.^30,31^ Structural and optoelectronic properties of low-energy structures found by CREST have then been investigated using (time-dependent) density functional theory simulations in a localized basis set framework, as implemented in the ORCA suite of programs.^32,33^ A complete account of the employed computational methods is reported in section S2 of the Supporting Information.

## Supporting Information Available

NEXAFS spectra measured using different techniques (section S1), detailed theoretical methods (section S2), fine analysis of NEXAFS simulations (section S3), fine analysis of structure–property relationships (section S4), Cartesian coordinates of optimized molecular structures (section S5), and transition from the gas phase to solution of ATP and ATPH^+^ (section S6) (PDF)
Transparent Peer Review report available (PDF)
