# Supplementary material for: Effect of Protonation on the Molecular Structure of Adenosine 5′-Triphosphate: A Combined Theoretical and Near Edge X-ray Absorption Fine Structure Study
Source: J Phys Chem Lett. 2023 Nov 5;14(45):10173–80. doi: 10.1021/acs.jpclett.3c01666 (PMC10658619; doi:10.1021/acs.jpclett.3c01666)
Supplement: Supplementary file 1 — jz3c01666_si_001.pdf [file jz3c01666_si_001.pdf]

Effect of Protonation on the Molecular Structure of Adenosine Triphosphate: a Combined Theoretical and Near Edge X-ray Absorption Fine Structure Study

Giuseppe Mattioli, Robin Schürmann, Chiara Nicolafrancesco, Alexandre Giuliani, and Aleksandar R. Milosavljević

Supporting Information

S1. NEXAFS spectra measured using different techniques.

S2. Detailed theoretical methods.

S3. Fine analysis of NEXAFS simulations.

S4. Fine analysis of structure-properties relationships.

S5. Cartesian coordinates of optimized molecular structures.

S6. Transition from gas-phase to solution of ATP and ATPH$^+$.

S1. NEXAFS spectra measured using different techniques.

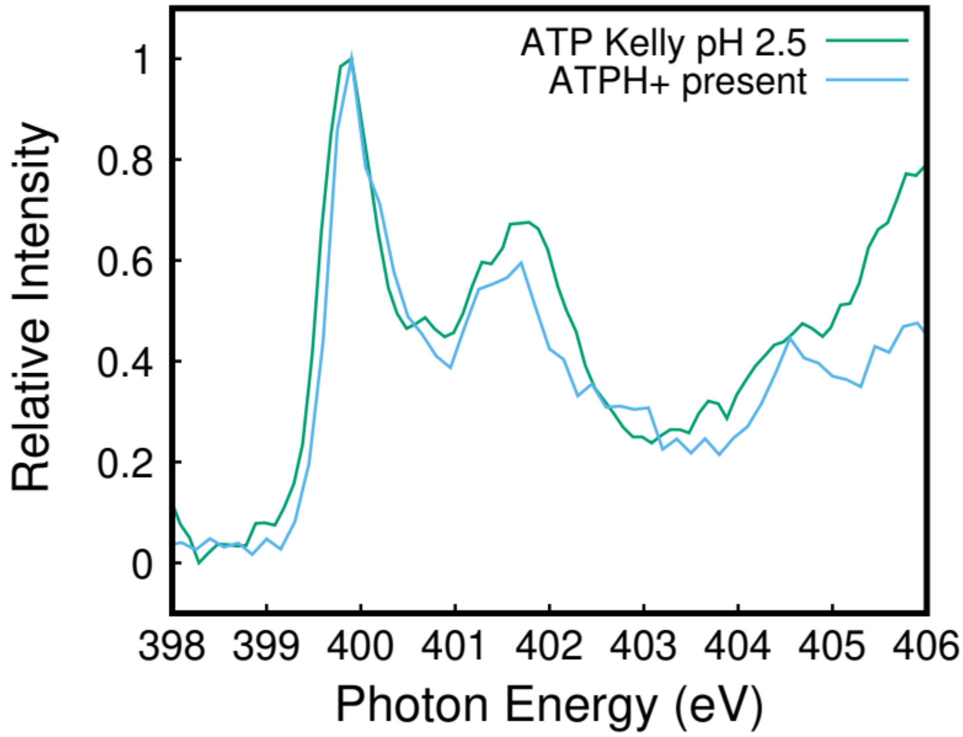

Figure S1. *N K-edge NEXAFS. Experimental photoabsorption spectra of isolated protonated ATP (light blue) and solvated ATP at pH=2.5 (dark green).*

In the main text, Figure 2a compares NEXAFS spectra at the N K-edge collected using different techniques. In particular, we discuss here the comparison between two spectra shown again in Figure S1, namely, ATP from acidic solution at pH = 2.5 (ATP_{aq}), measured using a liquid microjet apparatus (dark green plot in Figure S1) [1], and the present sample produced using an electrospray ionization source and stored in a linear ion trap (light blue plot). Both techniques are expected to yield samples mainly containing protonated ATP (i.e., $ATPH^+$), and we have already remarked in the main text the striking similarity between the spectra, that points to a close similarity to the samples. We want to add here a few technical details on the apparatus, that can explain minor discrepancies between the plots. The spectra were measured differently; the one of solvated ATP [1] was recorded as a total electron yield using a positively biased metal electrode placed close to the liquid jet/photon beam intersection, whereas the present one as a total ion yield (TIY) summed within the mass/charge (m/z) detection limit. It should be noted that due to the low m/z cut-off of the quadrupole ion trap (m/z 110) in the present experiment, some fragment ions are not detected. With increasing the photon energy, particularly above the core ionization potential, the formation of lower m/z ions is favored, which could lead to apparent decrease of the TIY. Also, the present NEXAFS of dilute ionic targets was recorded with somewhat lower resolution than the one from ATP_{aq} , thus some spectroscopic structures are less resolved. Finally, a small blue shift of the present spectrum with respect to the previous one cannot be discussed because the precision of the photon energy calibration for the ATP_{aq} NEXAFS was not reported, therefore such discrepancy could be experimental.

S2. Detailed theoretical methods.

In order to ensure the full reproducibility of our calculations, we report here a complete account of the employed computational methods. The properties of adenine, ATP and $ATPH^+$ have been investigated using a multilevel computational protocol, based on tight-binding and (time dependent) density functional theory atomistic simulations. A preliminary wide screening of the molecular configurations of ATP (and adenine) in gas phase has been performed by using a semiempirical tight-binding method rooted on the GFN2-xTB Hamiltonian, as implemented in the xTB suite of programs [2]. The global energy minimum and an ensemble of low-energy conformers have been identified using the conformer-rotamer ensemble search tool (CREST), which uses GFN2-xTB as engine to perform a complex combination of (meta)dynamics simulations and geometry optimizations, blended by genetic sorting and mixing of structures [3]. In the case of ATP, a specialized algorithm has been used to evaluate all possible protonation sites and sort their energies in order to find the most stable protomers [4]. This procedure indicated the four N atoms labeled N1-N4 in Figure S2 as possible candidates for protonation. The CREST algorithm has been then applied to the four resulting protomers to find their most stable conformers. The 10 lowest-energy conformers find by CREST for each molecule have been then investigated using DFT-based simulations in a localized-basis-set framework, as implemented in the ORCA suite of programs [5]. CREST structures have been fully re-optimized using the M062X functional [6], the D3 dispersion correction [7] and the def2-TZVPP Gaussian basis set [8] to calculate electronic energies, and using the r^2SCAN functional together with its tailored mTZVPP basis set [9] to calculate the zero-point vibrational energy. The conformers have been sorted in agreement with their E+ZPE values to find the most stable configuration of each system, used for the calculation of spectroscopic properties. Total energies of such most stable conformers have been also recalculated using a very accurate wavefunction-based coupled-cluster approach. In detail, the calculations have been performed at the CCSD(T) level of theory using a domain-local pairs of natural orbitals (DLPNO) approximation and the large def2-QZVPP basis set, as also implemented in ORCA [10]. The results confirm the ordering of protomers indicated by DFT calculations, strengthening the slight prevalence of protomer 3: the relative energies in eV are Protomer 3 = 0, Protomer 1 = +0.09, Protomer 2 = +0.15, Protomer 4 = +0.52. Finally, time-dependent DFT (TD-DFT) calculations, restricted to core levels of O, N and C as occupied orbitals, have been performed to calculate NEXAFS spectra using the BH&HLYP functional [11], and the mentioned def2-TZVPP basis set, computing up to 400 electronic transitions for each atomic species. NEXAFS vibrational structures discussed below have been calculated by using the independent-mode displaced harmonic oscillator (IMDHO) model, as implemented in the ORCA advanced spectral analysis (ASA) module. We did not present in this study the results of our NEXAFS calculations at the P L-edge, because they cannot be currently performed using the same TDDFT framework used for K edges, due to theoretical complications in the treatment of the spin-orbit interaction. This would have forced us to introduce and assess a different calculation method and we preferred to refrain from introducing further complexity in this letter.

S3. Fine analysis of NEXAFS simulations.

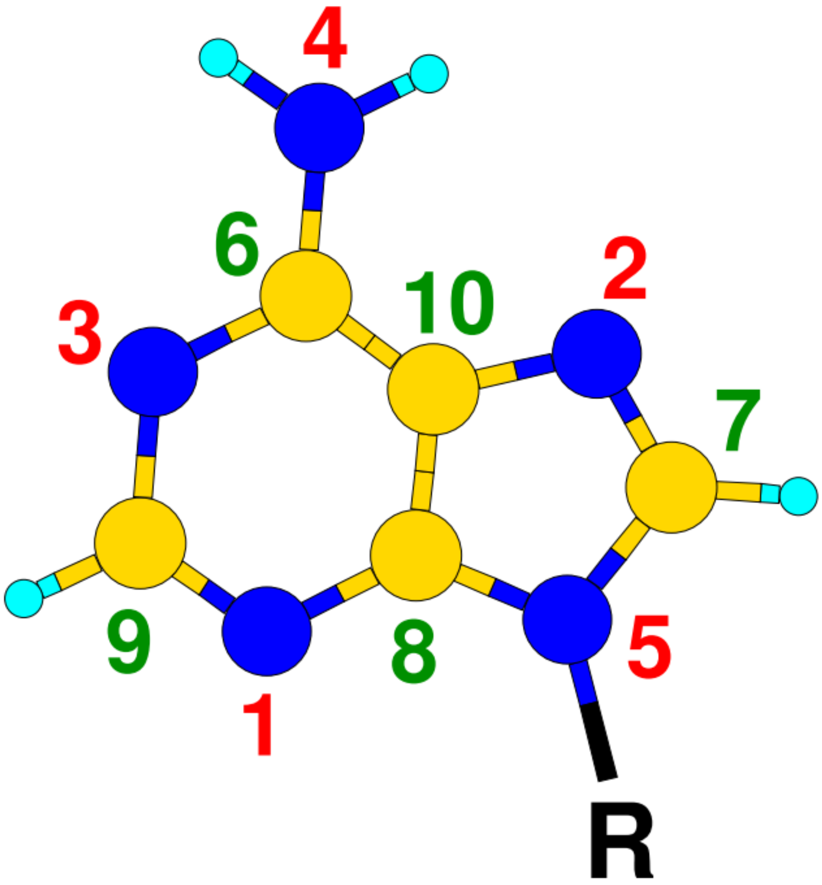

Figure S2. *Ball-and-stick model of the adenine unit of the investigated molecules. Numerical labels are assigned to nitrogen and carbon atoms to facilitate the discussion of results.*

Table S-I. *Lowest-energy NEXAFS transitions arising from 1s orbitals of the N1-N5 atoms labeled in Figure S2. In the table cells are reported transition energies, assignment to the N 1s donor orbital, and Oscillator strength (in a.u.). A * indicates the protonation site. The same shift of 2.7 eV has been applied to all results to favor the comparison with measurements.*

N K-edge	Adenine	ATP	Protomer 1	Protomer 2	Protomer 3
1	400.03 N2<br>0.057555	400.09 N3<br>0.069723	399.91 N2<br>0.066598	399.94 N3<br>0.064444	399.98 N2<br>0.057143
2	400.11 N1<br>0.053492	400.33 N1<br>0.061499	400.13 N3<br>0.061102	400.24 N1<br>0.057612	399.99 N1<br>0.061622
3	400.22 N3<br>0.058080	400.42 N2<br>0.053254	401.57 N4<br>0.036040	401.96 N4<br>0.025053	402.01 N4<br>0.028385
4	402.51 N4<br>0.021599	401.81 N4<br>0.023205	402.44 N1-H*<br>0.038211	402.13 N2-H*<br>0.041272	402.30 N3-H*<br>0.040804
5	403.02 N5<br>0.038722	402.87 N5<br>0.040359	403.24 N5<br>0.041009	402.43 N5<br>0.042393	402.68 N5<br>0.040251

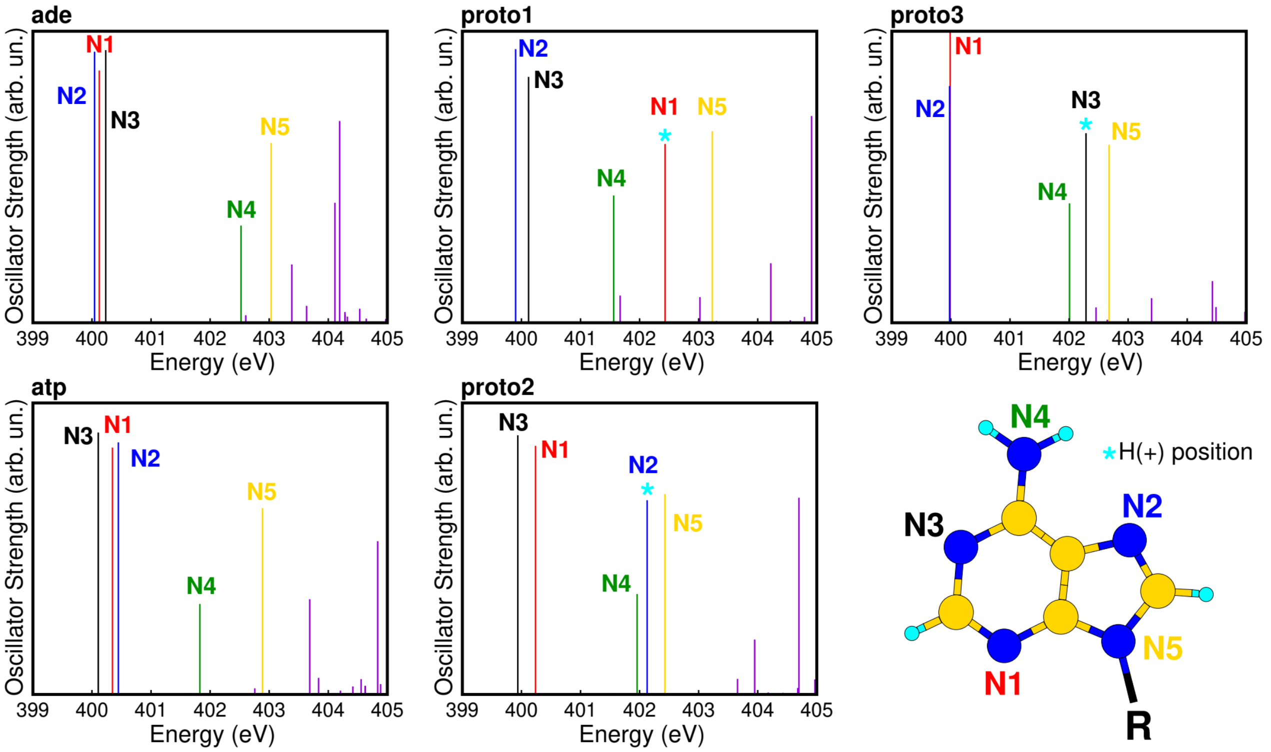

Figure S3. *N K-edge NEXAFS electronic excitations and corresponding oscillator strengths are reported as bars for adenine, ATP, and three ATPH+ protomers, where a * indicates the protonation site. The lowest energy transition arising from the five nonequivalent N atoms of the molecules, listed in Table S-I, are reported as colored bars. The same shift of 2.7 eV has been applied to all results.*

Numeric labels of N and C atoms of adenine and adenine units contained in ATP and $ATPH^+$ protomers are defined in Figure S2 and used hereafter. As the excess proton is attached to N atoms, we start the fine analysis of NEXAFS excitations based on TDDFT calculations from N spectra. The lowest five bound excitations of adenine, ATP and of the three $ATPH^+$ protomers discussed in the main text, corresponding to the first two features of N NEXAFS, are highlighted as colored bars in Figure S3 and also listed in Table S-I. As anticipated in the main text, the most prominent feature of the plots is represented by the blue shift of around 2 eV upon protonation of the corresponding transitions (cyan stars *), with respect to the same line in ATP, also accompanied by a slight reduction of its oscillator strength. Such blue shift is consistent with the relative reduction of intensity of the first peak of the spectrum, which in adenine and ATP is formed by three lines (N1, N2 and N3) close in energy, with respect to the second peak, which acquires the contribution of the protonated N. Moreover, as also discussed in the main text, the first core excitation arising from N4 is systematically red shifted in $ATPH^+$ (and ATP) with respect to adenine, contributing to the gap reduction between the first and second peak measured for $ATPH^+$. Minor rearrangements of the N4, N5 and protonated N in the second peak produce differences in the calculated vibronic spectra, reported in Figure S5 as Voigt convolutions of vibrational progressions. It can be noted that all protomers spectra are slightly red shifted with respect to adenine. More interestingly, protomer 1 is characterized by a broad distribution of the second block of transitions involving N1, N4 and N5, which is apparently less similar to measurements with respect to protomer 2 and 3.

Table S-II. *Lowest-energy NEXAFS transitions arising from 1s orbitals of the C6-C10 atoms labeled in Figure S2. In the table cells are reported transition energies, assignment to the C 1s*

donor orbital, and Oscillator strength (in a.u.). The same shift of 2.6 eV has been applied to all results to favor the comparison with measurements.

C K-edge	Adenine	ATP	Protomer 1	Protomer 2	Protomer 3
1	286.49 C9 0.07150	286.48 C9 0.07172	286.59 C9 0.07455	286.37 C9 0.07384	286.67 C9 0.07114
2	287.01 C7 0.06610	286.83 C7 0.06882	286.81 C7 0.06583	286.84 C7 0.07424	286.78 C7 0.06759
3	287.25 C6 0.06368	287.05 C6 0.07416	286.98 C6 0.07598	287.30 C6 0.06622	286.99 C10 0.03938
4	287.35 C10 0.06328	287.38 C10 0.05514	287.29 C10 0.05019	287.48 C10 0.05646	287.12 C8 0.07035
5	287.54 C8 0.08099	287.54 C8 0.07830	287.72 C8 0.07467	287.64 C8 0.07660	287.41 C6 0.09027

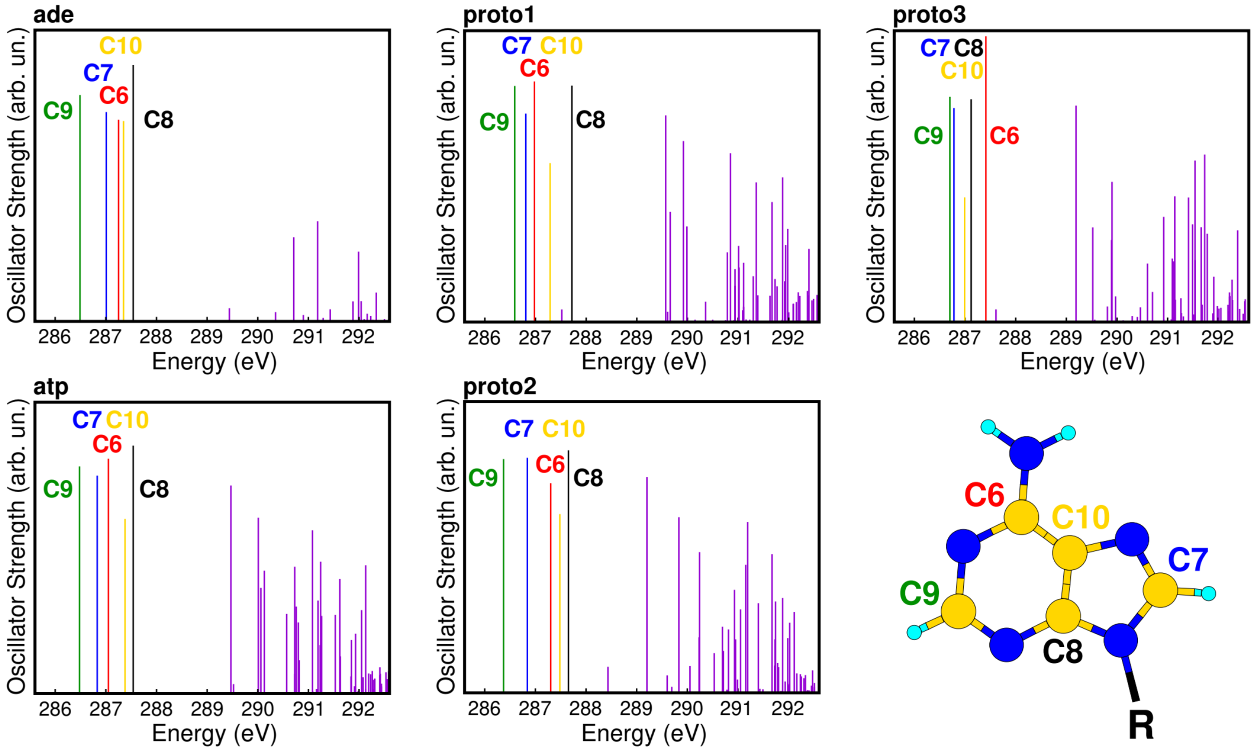

Figure S4. *C K-edge NEXAFS electronic excitations and corresponding oscillator strengths are reported as bars for adenine, ATP, and three ATPH$^+$ protomers. The lowest energy transition arising from the five nonequivalent C atoms of the adenine block of the molecules, listed in Table S-II, are reported as colored bars. The same shift of 2.6 eV has been applied to all results.*

C K-edge NEXAFS spectra present less obvious differences among them than N K-edge ones. However, the proximity between adenine C atoms and neighboring protonation sites on N atoms in a π-conjugate system is responsible for the modulation of C transitions, as shown in Figure S4 and in Table S-II, that may provide useful hints for a sounder assignment of the preferred protonation site. First of all, the first broad feature of the spectrum between 286 and 288 eV is wholly contributed by transitions arising from 1s orbitals of the adenine C atoms. This is expected, because core holes are better screened in conjugate rather than saturated systems such as those found in the ribose unit. Apart from a weak feature clearly visible above 290 eV in the adenine spectrum, which

is assigned to further electronic transitions involving the adenine C6-C10 atoms, the steady increase of the spectrum above 289 eV in the larger molecules is almost entirely due to the contribution of ribose C atoms, with negligible differences among ATP and $ATPH^+$.

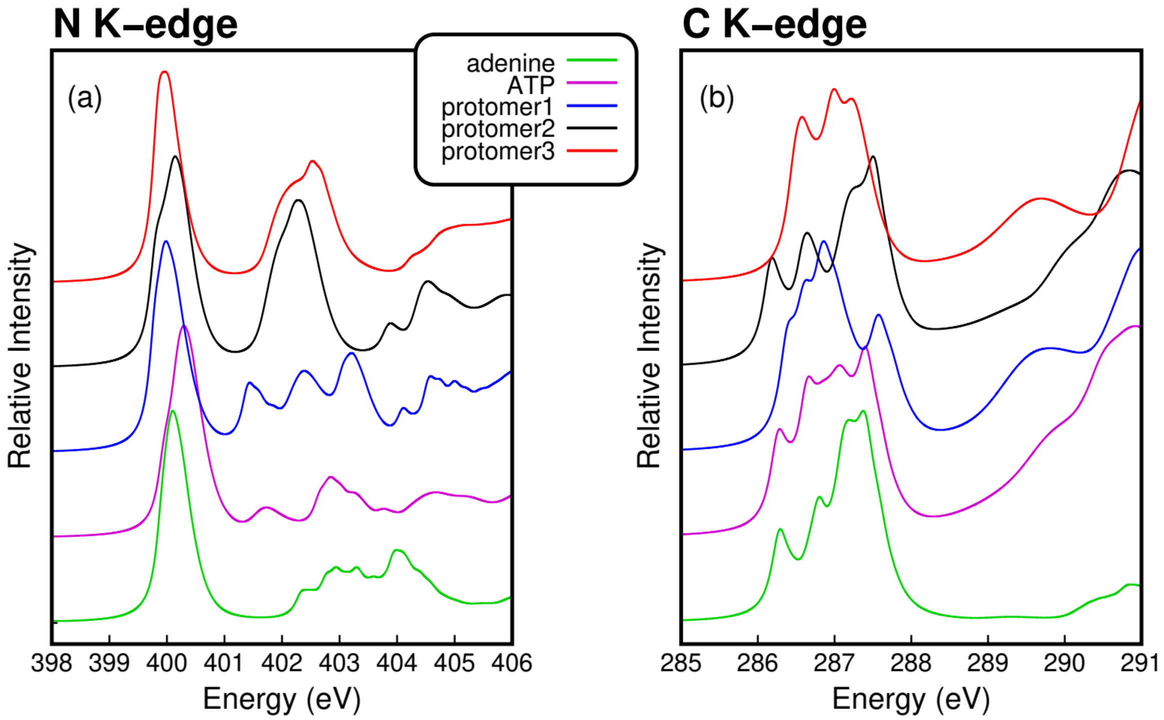

Figure S5. *Calculated N and C 1s NEXAFS spectra of adenine, ATP, and three* $ATPH^+$ *protomers, including contribution of vibrational states (harmonic approximation). All N (C) spectra have been shifted by 2.7 eV (2.6 eV) to favor the comparison with measurements.*

If we take a closer look to the first feature of the C spectra, assigned to the five lowest-energy strong electronic transitions of the C6-C10 atoms, we note that the colored bars in Figure S4 are ordered in the same way (C9, C7, C6, C10, C8) in all species but protomer 3, whose ordering is C9, C7, C10, C8, C6. The effect of the additional proton on the conjugate structure of the adenine unit is responsible for this inversion. To shed light on this mechanism we show in Figure S6 density-difference maps of four NEXAFS electronic transitions involving the C6 and C8 atoms in protomer 3 and protomer 1. In detail, the maps show the electronic density difference between the ground state and the excited state corresponding to a given electronic transition in the NEXAFS spectrum; in other words, charge is displaced from blue regions to red regions upon excitation of the molecule. Preliminarily, we note that blue regions are, as expected, strictly confined to atomic-like 1s orbitals, visible in transparency inside the red regions surrounding the C6 and C8 atoms (gray arrows in the figure). Then, let us focus on the role of excess protons in the transitions. When the core hole and the excess proton hold a reciprocal *ortho* position, (as in the case of C6*/N3-H^+ and C8*/N1-H^+) there is a repulsive strain of positive charges, which summons delocalized π-conjugate clouds to screen the charge. This process blocks the transposition of the excited electron through the C9=N1 (in the case of protomer 3) or C9=N3 (in the case of protomer 1) double bond, as shown by red arrows in Figure S6. This results in less delocalized final states for the excited electron and, in turn, in higher energy for the corresponding NEXAFS lines. When the core hole and the excess proton hold a reciprocal *para* position (as in the case of C6*/N1-H^+ and C8*/N3-H^+), on the contrary, the excited state is more delocalized across the whole six-membered pyrazine ring, in order to better screen the relatively farther positive charges, allowing participation of C9 in the charge

accumulation of excited states (green arrows) and resulting in lower energies for the corresponding NEXAFS lines and, in turn, in the inversion of the C6 and C8 lines in the spectra of protomer 1 and 3.

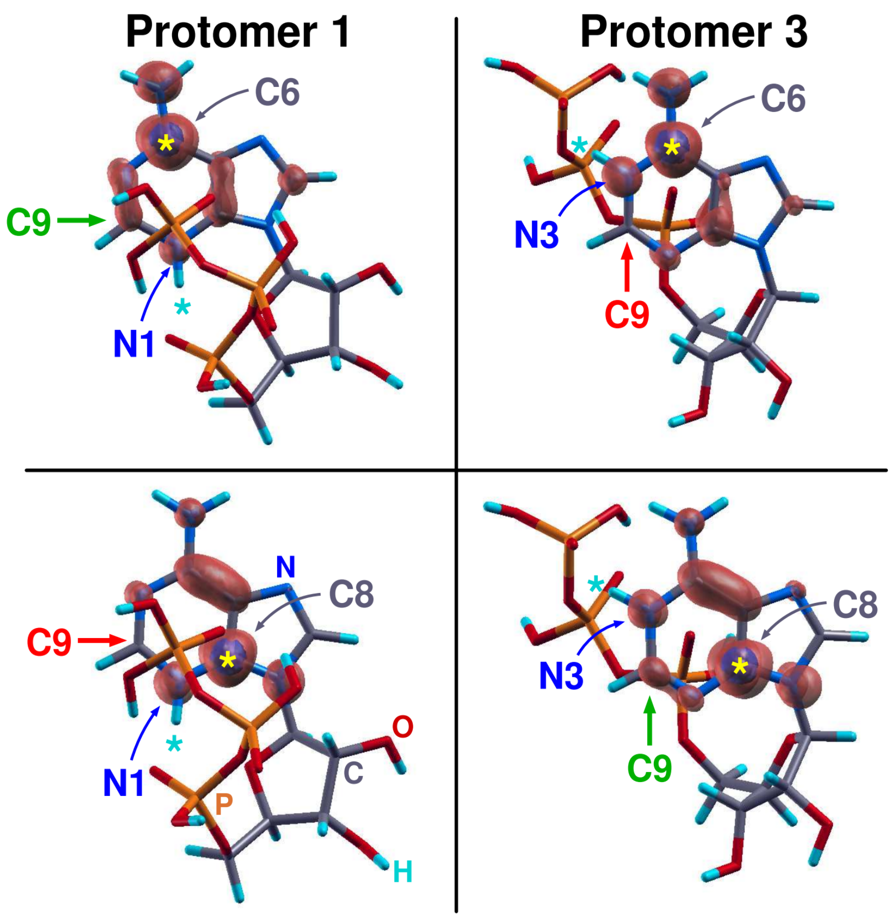

Figure S6. *Difference-density maps of four NEXAFS transitions arising in protomer 1 and protomer 3 from two carbon atoms labeled C6 and C8 in Figure S2. Charge density is displaced from blue regions to red regions upon excitation. Green and red arrows indicate allowed and blocked conjugation, respectively, of the C9 site, as discussed in the text. Yellow and cyan stars indicate the positions of core holes and excess protons, respectively.*

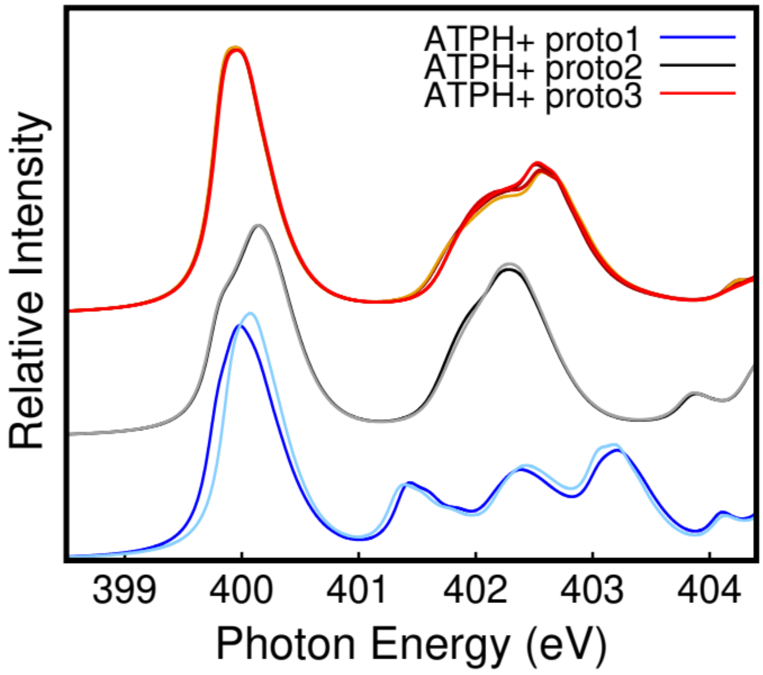

Figure S7. *Calculated N 1s NEXAFS spectra of eight lowest-energy rotamers of the three ATPH$^+$ protomers, including contribution of vibrational states (harmonic approximation). All spectra have been shifted by 2.7 eV to favor the comparison with measurements.*

Even if there is no conclusive argument to fully support the assignment, the cross comparison of the calculated N and C 1s vibronic NEXAFS spectra suggests that protonation of N3 yields spectra in an overall slightly closer agreement with measurements than N1 and N2. This also agrees with the energetics of the three protomers, slightly favoring protomer 3 with respect to protomer 1 and protomer 2. However, it cannot be excluded the concurrence of more protomers. Moreover, rearrangement of the same protomers between slightly different low-energy rotamers (as found by CREST and reoptimized at DFT level, within 1 eV from the most stable rotamer of protomer 3) has been considered. The calculated N 1s vibronic NEXAFS spectra related to all the eight considered rotamers, shown in Figure S7, are very similar one another and well distinguished among different protomers, but can all the same contribute to blend the results, preventing the unambiguous assignment of protomers in the measured sample.

S4. Fine analysis of structure-properties relationships.

In order to provide further insight into structure-properties relationships, Atoms-in-molecule (AIM) charges have been calculated using the Bader partitioning method [12]. In this case, 9-methyl-adenine has been also added to the investigated molecules, with the purpose of demonstrating that its charge density distribution is much more similar to ATP than adenine. It could therefore be considered as a more reliable reduced toy-model even for further experimental studies. The results are summarized in Table S-III. Some interesting trends can be highlighted by this results and cross-compared with calculated spectra (see above) and protonation maps (see below). First of all, protonation clearly affect not only the charge distribution on N atoms, but also the charge distribution on C atoms, as already indicated by the fine analysis of C 1s NEXAFS spectra. Moreover, protonation only induces a slight increase of charge on the adenine unit of $ATPH^+$ protomers with respect to ATP (around 0.1 a.u., localized on the newly formed N-H moieties), which is necessary to the screening of the positive charge of the added proton, accompanied by some charge displacement inside the same adenine block.

*Table S-III. Bader valence (i.e., 2s + 2p) atomic charges of (adenine unit) C and (all) N atoms of adenine, 9-methyl-adenine, ATP and three $ATPH^+$ protomers. Atomic labels are defined in Figure S2. Protonation sites are indicated by *.*

Bader valence atomic charge	**Adenine**	**9Me-adenine**	**ATP**	**Protomer 1**	**Protomer 2**	**Protomer 3**
**N1**	7.97	8.01	8.00	8.08*	7.99	7.99
**N2**	7.31	8.00	7.91	7.97	8.01*	7.95
**N3**	7.91	7.89	7.88	7.10	7.88	7.99*
**N4-H_2**	8.01	8.02	8.02	7.98	8.01	8.01
**N5**	8.02	7.84	7.87	7.89	7.84	7.92
**C6**	1.39	1.47	1.37	2.19	1.26	1.53
**C7-H**	2.23	2.26	2.25	2.27	2.23	2.23
**C8**	1.52	1.51	1.49	1.68	1.46	1.54
**C9-H**	2.22	2.17	2.19	2.17	2.18	2.21
**C10**	3.42	2.70	2.85	2.61	3.04	2.58
**Total (incl. H)**	50.00	49.87	49.83	49.94	49.90	49.95

The main relocation of charge induced by protonation is discussed with the help of Table S-III and of protonation maps in Figure S8. For the sake of clarity, the latter maps have been obtained in the case of 9-methyl-adenine, having a charge distribution inside the adenine unit close to ATP. A single proton has been added to the structure without structural relaxation and the maps display the charge density difference between the protonated and unprotonated structures, with the charge density displaced from blue regions to red regions upon protonation. We stress that only the added proton is relaxed after its addition to the structures; this is necessary to calculate the maps, that can, therefore, only show tendencies, while charges in Table II correspond to fully relaxed systems so that the information must be cross compared. In protomer 1 the main displacement involves a net charge transfer from N3 to C6. This can be interpreted using resonant structures typical of reaction intermediates of electrophilic aromatic substitution reactions [13]: the positive charge induced in N1 by protonation can be partly delocalized in the ortho (C8 and C9) and para (C6) position of the 6-

terms aromatic ring, where it is screened through delocalization of the aromatic charge. This is particularly visible in the case of the C6 atom (the blue spot, having an evident π character, see the green arrow in Figure S8). However, the C6 atom is surrounded by two electron rich neighbors, namely the N3 -aza- and the terminal N4-H_2 group, both bearing a lone pair. N4 reacts to the lack of charge by assuming an sp^2 hybridization that does not alter its total charge. The C6-N4 bond shrinks from 1.34 Å in ATP to 1.31 Å in protomer 1, with a significant increase of the Mayer bond order from 1.19 (almost single bond) to 1.76 (almost double bond). On the other hand, there is an elongation of the C6-N3 bond from 1.33 Å in ATP to 1.35 Å in protomer 1, corresponding to a decrease of the Mayer bond order from 1.41 to 1.24, suggesting a partial relocation of the aromatic bond on C6. As a final remark, it can be noted that protomer 3, so far the best $ATPH^+$ candidate, is characterized by the closest distribution of electronic charge to ATP.

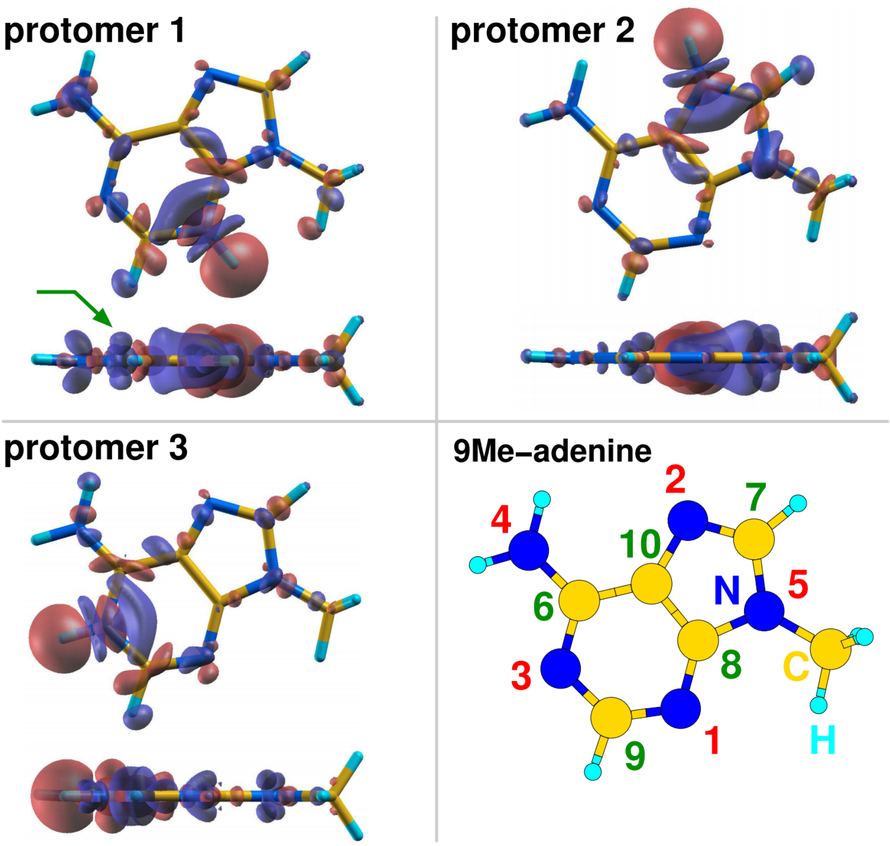

Figure S8. *The maps (top and side views) display the charge displacement induced in 9Me-adenine (preferred to adenine because its charge density distribution is more similar to ATP) by the addition of a proton. Charge is displaced from blue regions to red regions upon protonation of N1, N2 and N3 nitrogen atoms, as reported in the ball-and-stick model.*

S5. Cartesian coordinates of optimized molecular structures.

We make available the Cartesian coordinates of the five molecular structures (adenine, ATP and three $ATPH^+$ protomers) on which NEXAFS spectra have been calculated. The optimized structures of ATP and $ATPH^+$ protomers are also shown in Figure S9.

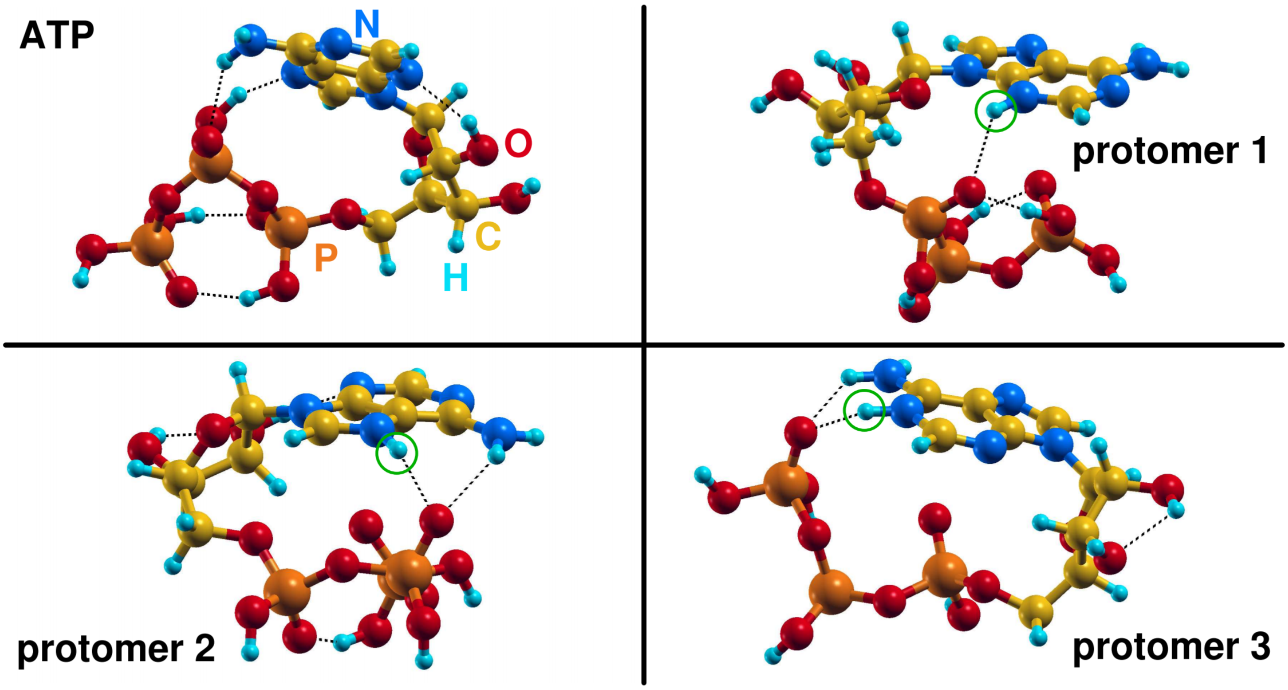

Figure S9. *Geometries of ATP and of the three* $ATPH^+$ *protomers investigated in this study, optimized using the dispersion-corrected M06-2X functional and the def2-TZVPP basis set. Additional protons are enclosed in green circles. H-bonds, shown as dashed lines, promotes the formation of a coiled molecular structure in gas phase.*

Adenine

N	-1.65291283449768	0.78633977149086	-0.06188589938479
N	-5.34125130521171	-0.68765530037561	-0.01850936547056
N	-2.54019267311875	2.90523189921657	-0.19287199486031
N	-2.97318939171083	-1.18956656666539	0.03625885843408
N	-5.24669062086027	1.52420786106534	-0.14392424697759
C	-2.71211061239069	1.58012433203563	-0.11912939415455
C	-1.83841951606846	-0.52550537229511	0.01096645275595
C	-4.01609853104382	-0.37762247581428	-0.02185001645912
C	-3.97897347301136	0.99818592394126	-0.10017715802743
C	-6.01955217733680	0.49461290398466	-0.09341229941469
H	-7.08976342547519	0.52454696764257	-0.10657152554295
H	-0.94107979276089	-1.11687898369640	0.05456119980406
H	-3.32887606044892	3.50876472733636	-0.23428875782444
H	-5.72931389374745	-1.60352739381117	0.02987402898179
H	-1.61847569231714	3.27710170594471	-0.20239988185945

ATP

P	-3.90060735530530	2.20019412046450	-0.32301049967183

P	-0.45121176052546	1.80608314800556	-0.34805028739258
P	-2.40777150431619	-0.35619903587651	-0.15265343222307
O	-1.11556445413150	0.41585972544056	-0.69272385402050
O	-3.58569739748963	2.63639461530932	1.11643579829264
O	-3.59490115443574	0.64222729377161	-0.39393871632305
O	3.07588395921130	0.64399331991835	1.49483590506296
O	5.39619987635819	1.13119228738831	-0.32974398800089
O	1.06199962371503	1.45470332550387	-0.30808476941591
O	4.47662543834966	-1.20681949552274	-1.13547853076348
O	-2.56546502937585	-1.61339267074380	-0.83932707172195
O	-0.61660838628308	2.67325810826072	-1.61271680483499
O	-3.21907698947658	2.90127201469145	-1.40545238752668
O	-2.28683109267781	-0.39830996360520	1.37954898535484
O	-0.97634585800093	2.37301599622070	0.89049457253891
O	-5.45105279815945	2.25111099776795	-0.38240023626834
N	0.70209333552126	-4.63988579112018	-0.75074965200863
N	2.56471873661416	-3.22439667507454	-0.38065015994776
N	2.03404747473279	-1.37218332469130	1.06557616872786
N	-0.08508468443286	-1.82621126877691	1.48894099371454
N	-1.41955025372281	-4.21615035304536	0.02414318106491
C	1.98901515535259	2.39003738486466	0.25913648569610
C	1.74779023895980	-2.51985211636507	0.39080642074330
C	-0.12283711779968	-3.88781475241240	-0.02744498346992
C	3.28990676394439	1.70108805652838	0.56853346551578
C	0.42191687253457	-2.78882917429574	0.64622508106698
C	3.24754301616030	-0.59376812097711	0.89380123910307
C	1.96403415744616	-4.28476282634724	-0.88577824135118
C	3.52623156221363	-0.35130949890450	-0.59717253565949
C	4.02777902674851	1.08091837678560	-0.60469189877294
C	0.89993490130943	-1.01143733178644	1.70238806249905
H	-2.62100458021809	2.64626933416076	1.28262602476605
H	-1.69037360342225	-4.93067111381271	-0.61825511495547
H	2.57029031006369	-4.92881858250947	-1.49752213315125
H	-5.79821477361489	2.53857652189238	-1.22083189738823
H	-1.55171319210845	2.94154484023680	-1.73119493852566
H	-1.56766579096625	-1.03437550960835	1.63787026473014
H	5.80768842802799	0.37876908640114	-0.74611802473399
H	4.12350582075915	-2.09856219096179	-1.08078381307445
H	3.93622613102305	2.43134112038365	1.03936651513468
H	3.79991768658451	1.58211081536801	-1.54011989715606
H	4.07230843734931	-1.12455777685102	1.35299732113532
H	0.84946325880713	-0.12441982872168	2.29540922848478
H	1.56373477197138	2.78833849682462	1.16974737263720
H	-2.07109752989698	-3.46063992834622	0.11536613130610
H	2.59014765551730	-0.40312294325646	-1.14019488614591
H	2.15121805468436	3.19712076612387	-0.44441594447105

Protomer 1

P	-2.77002210748976	0.20147134221710	0.26011274977445

P	-0.18855706028501	-2.23263414649598	-1.17986979262068
P	-1.37520741571941	-1.99448045828182	1.39927391777643
O	-0.23104389788357	-1.70325112302495	0.31620206905338
O	-4.21750672468694	0.62301475091772	0.58947053797609
O	-2.64432022816704	-1.32390165765020	0.73203701546650
O	2.17303142649382	-0.00465738461348	-1.00965535616568
O	4.65794563611027	-1.73062136314973	0.55008385283953
O	1.23865645850920	-2.79143622959732	-1.34256212152305
O	3.59347878755100	0.03602712663229	2.22207123796705
O	-1.54040913425858	-3.40748352814668	1.60921024861380
O	-1.12798772271463	-3.45281273196264	-1.21021194980896
O	-1.75993004747168	0.98447909894198	0.95516158383125
O	-0.96786780410297	-1.10067294607488	2.58214477175908
O	-0.55981961544770	-1.16053646486012	-2.10225760045624
O	-2.71675008545357	0.12988629397942	-1.27999023955987
N	-1.45010977898652	3.47818255502367	-1.51210103913227
N	0.11006962053408	1.74408802819550	-1.54504772347729
N	1.60886787254797	1.71959743530670	0.40035263156200
N	0.76942688480803	3.50894723799406	1.42224286596133
N	-1.55782146308026	4.98513868936561	0.20535423596257
C	2.35872969962584	-2.17232579617530	-1.96787360213871
C	0.62329674397772	2.18930595794120	-0.38408132761342
C	-0.97645680819508	3.94184244278254	-0.33940850194262
C	3.00732648319017	-1.15307210843116	-1.08201777636761
C	0.11119090269596	3.29088921466431	0.24849722101305
C	2.59110990595175	0.70253996484089	0.11188793517503
C	-0.92336052897744	2.43232954319769	-2.04819131383446
C	2.76113343960477	-0.35721991858109	1.19308466381450
C	3.27934668670892	-1.56329456254730	0.37269367750289
C	1.64821623917643	2.57421501236291	1.48454115548910
H	-4.92138890703024	0.27654438900536	0.04745324946215
H	-2.33149998260539	5.41604519563788	-0.25333143177433
H	-1.32659405748894	2.05893712301949	-2.97030439243761
H	-1.96883431772853	-0.36286438777365	-1.68386859025243
H	-1.29274690875003	-3.91620924527619	-0.37934869708942
H	-1.09472127709583	-0.15809402043301	2.39769910563193
H	4.84539633107300	-2.50660115784919	1.06386630640615
H	0.40210267532634	0.87292479416928	-1.95171428992516
H	3.95804123179264	-0.87740778199359	-1.52943089408218
H	2.74285146700473	-2.46167781955881	0.63914622131910
H	3.54591803883458	1.19051533016048	-0.07594835283123
H	2.36340188005887	2.42437948580961	2.26729324863189
H	2.04763411855169	-1.72091787858877	-2.89960549177317
H	-1.23547955419484	5.34166234240069	1.07949340039360
H	1.78790508830216	-0.58487415253975	1.60568419129653
H	3.05179687235767	-2.97269772748645	-2.17659033004401
H	4.48476421802623	-0.24052630187434	2.01989991347102

Protomer 2

P	-0.49327410483347	1.57460642358419	-1.82438009767954
P	2.48441990458806	0.95154496726636	0.06058117678647
P	0.30515410651677	2.87997096940456	0.62628336604946
O	1.36618510508317	1.73155747607382	0.86373339728340
O	-0.53370219566985	0.38115861541547	-1.01011215184757
O	-0.06320004277191	2.82636186758679	-0.89288014615499
O	1.22632688567519	-2.84959873105505	1.54731000503271
O	1.81781506724084	-4.74785636566467	-0.70701688908183
O	2.11577643529563	-0.51093321111180	0.37925180817207
O	-0.51455650576704	-3.73195560992200	-1.37350550594420
O	-0.78522078017284	2.73811881364580	1.56498420772263
O	2.55459594212857	1.36113905018949	-1.33086733199513
O	-1.85401204904393	1.99257161086055	-2.42864022012472
O	1.07368620771672	4.21620499505967	0.75601429253591
O	3.79108039980670	1.19639874923384	0.87126178180158
O	0.50170287387011	1.65749473197707	-2.99841356777946
N	-4.13888766931007	-0.27479647324727	-0.25143470525563
N	-2.69259304795466	-2.15227081775928	-0.18270413464591
N	-0.81540353303824	-1.82081607959240	1.29500907550735
N	-1.32694326978438	0.13290822425854	2.05956159841999
N	-3.75740915525157	1.68139851971428	0.88889274616133
C	3.04805088026104	-1.57466318130810	0.64276547198581
C	-2.01511902092034	-1.47289320678900	0.71812283177893
C	-3.42376953985440	0.42144955187446	0.61895455081993
C	2.32002283726377	-2.89230231993390	0.63294062968874
C	-2.33261689606766	-0.23049387252585	1.19626035958505
C	0.01612864797619	-2.95765766219894	0.88856356477532
C	-3.75856921943571	-1.48990114332581	-0.59680918544185
C	0.29205533789167	-2.89466034575285	-0.62065313744756
C	1.73772752832300	-3.35801158893432	-0.69146435233590
C	-0.43444645568997	-0.82681229072322	2.07438100296260
H	-1.15984047497975	1.06896691729057	2.39258243181521
H	-4.52119980891829	2.07588707312337	0.38375518542192
H	-4.38201052021114	-1.99017458410391	-1.31491702084611
H	1.41470511820534	1.50762415346360	-2.70150077777450
H	-1.84443124152005	2.52093887338707	-3.22215674621147
H	1.46226017684706	4.59542905451474	-0.02693076890946
H	1.12959361564486	-5.08899478083098	-1.27190684549397
H	-1.42358021313279	-3.44464976511247	-1.28803905856987
H	3.01747045559250	-3.64367685634942	0.98075516034930
H	2.25429754134368	-2.92268769136150	-1.54148391908365
H	-0.50224636298988	-3.86724904224420	1.16120264664753
H	0.47975937809675	-0.81521317701733	2.62153582626716
H	3.48524535349671	-1.39672850557419	1.61413619982985
H	-3.12370039967603	2.29501294384329	1.35489204213089
H	0.21923671717894	-1.86394033548551	-0.94577721067608
H	3.82903159416050	-1.57756542022221	-0.10553026191216
H	4.43058422239016	1.78214519617874	0.47560616458044

Protomer 3

P	3.81255975999568	-0.71798488045750	0.52723828126428
P	0.09505024164273	1.40624923834565	-0.84213921344471
P	2.85375244301465	1.81628943950996	-0.45258023680052
O	1.35829878062146	2.21975368530796	-0.26253160726089
O	5.30465273575492	-0.36199826429163	0.78779403000376
O	3.07237207646335	0.69060229689658	0.63268506900316
O	-3.12608372915440	0.51816288080807	-1.11149921027916
O	-4.55108697172147	2.15004921688362	1.65546176914569
O	-1.03909313785643	2.06433244172550	-0.04220489135109
O	-5.74204956179744	0.05537634616195	0.51064196153941
O	3.21843739430873	1.36154993201428	-1.78365945433992
O	-0.08848453404269	1.94053810531060	-2.29214062272760
O	3.25986112062501	-1.68501554106004	1.46252989922399
O	3.59725386742205	3.05134781646802	0.10391335285342
O	0.33187410520625	-0.01338201435622	-0.70698799239725
O	3.70570929810896	-1.12799889123038	-0.95208834268887
N	0.58437839404734	-2.03856860795108	1.58636409521437
N	-1.49550124995332	-0.98481059706673	1.55043421486612
N	-2.76113494120490	-1.64773942319206	-0.39966592959180
N	-1.38443536889644	-3.19779891004169	-1.22007611761687
N	1.34740547092884	-3.55749188338389	0.02087610826862
C	-2.25584437038024	2.64768712582825	-0.53012080437962
C	-1.71898351855453	-1.72273262171561	0.45803464745845
C	0.38077481627114	-2.80388913757172	0.49647678900169
C	-3.40417467619136	1.69396732937540	-0.38590817591572
C	-0.88150177580177	-2.68249504601915	-0.06456807935801
C	-3.74485307031124	-0.59713322882430	-0.52256698664468
C	-0.33756865720472	-1.17608421274353	2.06510491503458
C	-4.38603634626123	-0.12015634219824	0.77958647340228
C	-3.70600123917815	1.22836549636140	1.02694736535311
C	-2.48745750941967	-2.55728544601665	-1.39013791772010
H	5.68162449417218	-0.69985566771259	1.59505790029183
H	2.28214349890170	-3.45722573196313	0.35974112395622
H	-0.03636168947606	-0.62393970525186	2.93490808922442
H	3.61047626899391	-0.39437376171412	-1.58253512917258
H	0.32294505583353	1.42147245993418	-2.97799465094362
H	4.30355895385817	3.39457893050350	-0.43716253145609
H	-4.37289652748038	2.19154817071780	2.58659628916482
H	-6.06671081332480	0.77943508404538	1.03967967809702
H	-4.28748244881149	2.18928582960427	-0.78177825469596
H	-2.79076739130716	1.07986913059518	1.57909929695281
H	-4.52075528506799	-0.97726271624012	-1.17203096648969
H	-3.16076533638975	-2.68887048666773	-2.21258880920297
H	-2.12211188107332	2.93527413557909	-1.56125689419940
H	1.18531677923442	-4.05685758970919	-0.82753424242792
H	-4.23100725455332	-0.82158743842986	1.59041384475816
H	-2.43280969130467	3.52801484824553	0.06952160247884
H	1.53919363651400	-1.96525956381313	1.92007643084793

S6. Transition from gas-phase to solution of ATP and ATPH+.

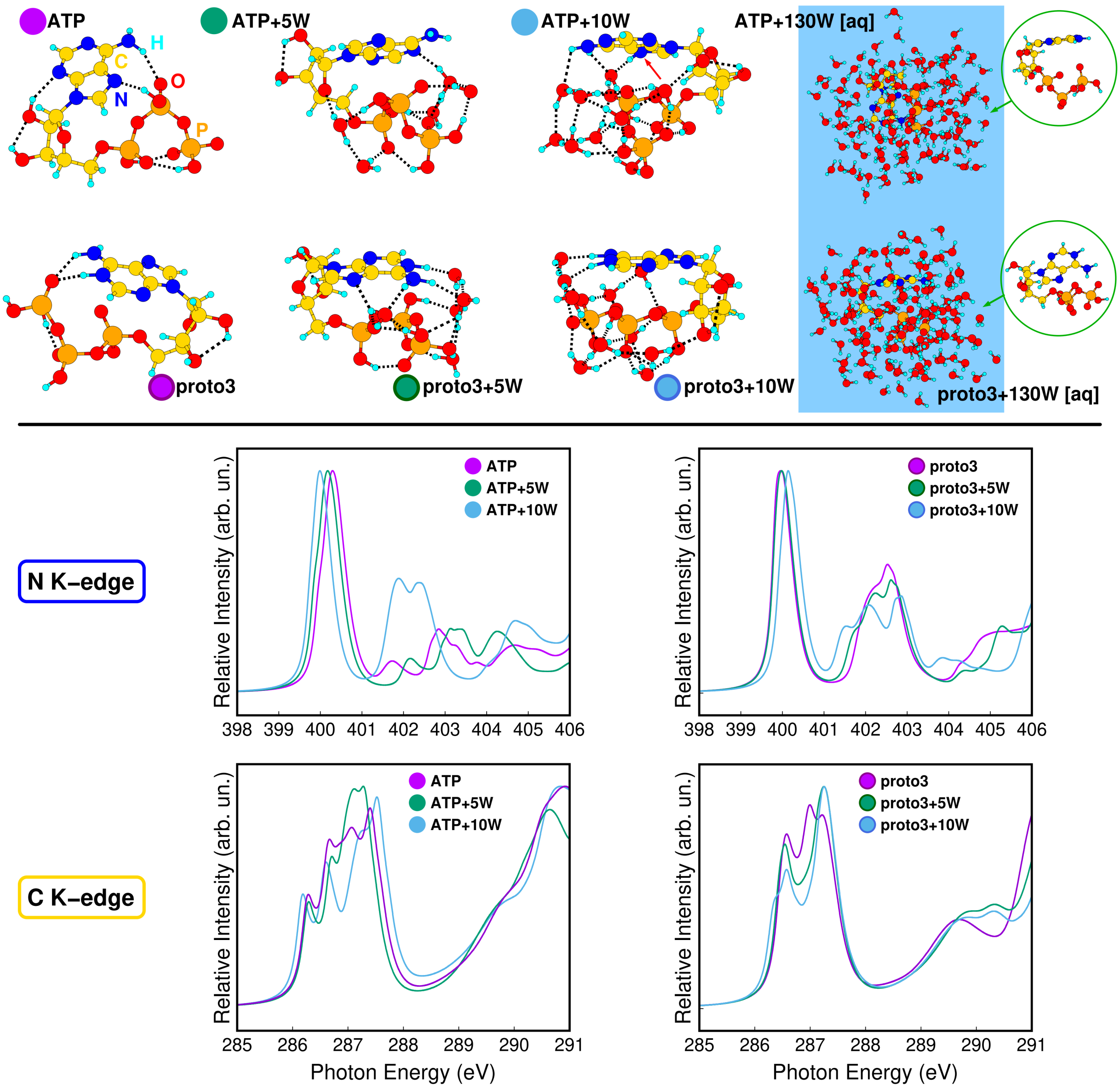

Figure S10. *Upper panel: Lowest-energy structures of ATP and of the ATPH+ protomer 3 in gas-phase clusters containing no, five or ten water molecules, and of the same molecules in water solution, surrounded by 130 explicit water molecules and immersed in an implicit solvent. In these last cases the coiled structure of the molecules alone are shown inside the green circles. A red arrow indicates the protonation of the N2 site in the ATP+10w system, as discussed in the text. Hydrogen bonds in gas-phase structures are represented by black dashed lines. Lower panels: Calculated C and N 1s NEXAFS spectra, including the contribution of vibrational states, of the six gas phase structures shown in the upper panel.*

The close similarity between the N 1s NEXAFS spectra of protonated ATPH+ obtained using different techniques (discussed in detail in Section S1) has stimulated further theoretical studies to unravel at what extent the presence of water molecules can affect present structural and spectroscopic results. It is no doubt that the present Near edge x-ray absorption mass spectrometry technique is able to select only protonated ATPH+, with no contribute of water molecules. The same cannot be considered as necessarily true in the case of the pressurized-jet technique used by Kelly et

al. [1], which uses a water solution at controlled pH as starting sample. Moreover, it has to be also considered that ATP is generally found as dianion in water solution at neutral pH, with deprotonation of the phosphate units [14], and that at lower pH the adenine unit can be protonated even before the phosphate units. Finally, water might heavily alter the strong tendency of neutral and protonated ATP to form coiled structures with the formation of strong H bonds between the adenine and phosphate units discussed in the main text and also shown in Figure S9.

To shed light on these hypotheses, we have performed a series of calculations that address the multiple role played by water, comparing the effects of the addition of different amount of H_2O molecules to neutral ATP and to the most stable $ATPH^+$ protomer 3. In detail, we have studied step by step a transition from the clusterization of a few water molecules together with the biomolecules in gas phase to the complete solvation of the same molecules in a water solution. The addition of water molecules has been controlled using a special version of the CREST algorithm discussed in Section S2, termed quantum cluster growth (QCG) [15], which has been used to surround ATP and $ATPH^+$ with a fixed amount of water molecules and to explore minimum energy structures, which are shown in the upper panel of Figure S10. Regarding the structural properties, first of all we note that, irrespective of the amount of water molecules, ATP and $ATPH^+$ retain the coiled structure found in gas phase. Water molecules preferentially interact with the polar phosphate units without entering the biomolecule pocket. In all structures, single water molecules generally act as bridges between phosphate and adenine, because this position probably reduces the strain required to bend the structures and form head-tail coils. We have already found at this stage a difference between ATP and $ATPH^+$: 10 water molecules in gas phase are sufficient to induce the deprotonation and ensure the screening of one of the phosphate groups in neutral ATP. The released proton is then preferentially localized on N2 in these conditions (red arrow in Figure S10), suggesting that gas-phase interactions may lead to unexpected localization of excess protons that cannot even be predicted on the grounds of well assessed properties of solutions. No shifting of protons is induced in the positively charged $ATPH^+$ in gas phase, in the presence of 5 or 10 water molecules. When a realistic water solution of ATP and $ATPH^+$ is simulated by raising the amount of explicit water to 130 molecules embedded in an implicit water solution, stable structures found are phosphate dianions, in agreement with the catalytic role of divalent cations as Mg^{2+} in the release of phosphate units in living organisms [14,16]. However, protons released in solution from phosphate units are not captured by nitrogen in the case of ATP, and don't alter the protonation of N3 in the case of the $ATPH^+$ protomer 3. Remarkably, in both solvated systems the molecules retain a similar coiled structure, as shown in Figure S10 in the two insets enclosed in green circles. Such results suggest that the protonation of the adenine unit in low-pH solutions is not affected by the transition from solution to gas phase obtained using different methods such as liquid microjet or electrospray ionization.

The analysis based on the properties of low-energy structures is strengthened by the results of N and C 1s NEXAFS calculations of the six gas-phase ATP and $ATPH^+$ structures shown in the upper panel of Figure S10. The spectra are shown in the lower panels of the same figure. Regarding the N K-edge, in the case of the neutral ATP molecule the addition of five water molecules partially in contact with N atoms through H-bonds does not significantly alter the N 1s NEXAFS spectrum, inducing a red shift of 0.1 eV of the main, sharp peak which is smaller than the uncertainty of experimental photon energy scales used in experiments (see Section S1). As discussed above, the addition of 10 water molecules promotes the formation of $ATPH^+$ in gas phase through deprotonation of phosphate, inducing a dramatic change in the shape of the spectrum and a larger red shift of 0.3 eV of the main peak, in line with the results obtained for the conventional $ATPH^+$ protomers in absence of water, shown in Figure 2d of the main text. The high compatibility of our calculated N 1s NEXAFS spectra of ATP in gas phase with the measured spectrum shown in Figure

2c, corresponding to a jet-spray sample from a pH=7.5 solution, suggests a secondary role of water in the spectroscopic measurements at the N K-edge and excludes the presence of higher amounts of water interacting with the molecule capable of inducing the migration of protons from phosphate to adenine through water molecules. The presence of water is even less significant in the case of the N 1s NEXAFS spectra of the protonated $ATPH^+$ protomer 3. The spectrum is almost insensitive to the addition of 5 water molecules, and only moderately blue-shifted (0.2 eV) in the case of the addition of 10 molecules, in both cases without strong alterations of its shape. Even less differences are found in the case of the C K-edge spectra upon addition of the same amounts of water molecules; the spectra of hydrated ATP and $ATPH^+$ are not shifted and only slightly modified in shape. Putting together all the information extracted from such calculations and from their comparison with measurements, we can also rationalize the close similarity between the two spectra shown in figure S1, concluding that both ATP and $ATPH^+$ have a strong tendency to form similar coiled or folded structures in gas phase and in solution, and that the presence of small amounts of water molecules connected to ATP and $ATPH^+$ through H-bonds does not significantly affect this tendency nor the electronic environment of N and C atoms, as reflected by calculated and measured NEXAFS spectra.

References.

1 Kelly, D. N.; Schwartz, C. P.; Uejio, J. S.; Duffin, A. M.; England, A. H.; Saykally, R. J. “Communication: Near Edge x-Ray Absorption Fine Structure Spectroscopy of Aqueous Adenosine Triphosphate at the Carbon and Nitrogen K-Edges.” *J. Chem. Phys.* **133**, 101103 (2010).

2 Grimme, S. “GFN2-xTB−An Accurate and Broadly Parametrized Self-Consistent Tight-Binding Quantum Chemical Method with Multipole Electrostatics and Density-Dependent Dispersion Contributions.” *J. Chem. Theory Comput.* **15**, 1652−1671 (2019).

3 Pracht, P.; Bohle, F.; Grimme, S. “Automated exploration of the low-energy chemical space with fast quantum chemical methods.” *Phys. Chem. Chem. Phys.* **22**, 7169−7192 (2020).

4 Grimme, S. “Exploration of Chemical Compound, Conformer, and Reaction Space with Meta-Dynamics Simulations Based on Tight-Binding Quantum Chemical Calculations.” *J. Chem. Theory Comput.* **15**, 2847−2862 (2019).

5 Neese, F. “The ORCA program system.” *WIREs Comput. Mol. Sci.* **2**, 73−78 (2012); Neese, F. “Software update: the ORCA program system, version 4.0.” *WIREs Comput. Mol. Sci.* **8**, e1327 (2018).

6 Zhao, Y.; Truhlar, D. G. “The M06 suite of density functionals for main group thermochemistry, thermochemical kinetics, noncovalent interactions, excited states, and transition elements: two new functionals and systematic testing of four M06-class functionals and 12 other functionals.” *Theor. Chem. Acc.* **120**, 215−241 (2008).

7 Grimme, S.; Antony, J.; Ehrlich, S.; Krieg, H. “A consistent and accurate ab initio parametrization of density functional dispersion correction (DFT-D) for the 94 elements H-Pu.” *J. Chem. Phys.* **132**, 154104 (2010).

8 Schäfer, A.; Horn, H.; Ahlrichs, R. “Fully optimized contracted Gaussian basis sets for atoms Li to Kr.” *J. Chem. Phys.* **97**, 2571−2577 (1992).

9 Grimme, S.; Hansen, A.; Ehlert, S.; Mewes, J.-M. “r^2SCAN-3c: A “Swiss army knife” composite electronic-structure method.” *J. Chem. Phys.* **154**, 064103 (2021).

10 Riplinger, C.; Neese, F. “An efficient and near linear scaling pair natural orbital based local coupled cluster method.” *J. Chem. Phys.* **138**, 034106 (2013).

11 Becke, A. D. “A new mixing of Hartree-Fock and local density functional theories.” *J. Chem. Phys.* **98**, 1372 (1993).

12 Tang, W.; Sanville, E.; Henkelman, G. “A grid-based Bader analysis algorithm without lattice bias.” *J. Phys.: Compute Mater.* **21**, 084204 (2009); Sanville, E.; Kenny, S. D. ; Smith, R.; Henkelman, G. “An improved grid-based algorithm for Bader charge allocation.” *J. Comp. Chem.* **28**, 899−908 (2007); Henkelman, G.; Arnaldsson, A.; Jónsson, H. “A fast and robust algorithm for Bader decomposition of charge density” *Comput. Mater. Sci.* **36**, 254−360 (2006).

13 Castrovilli, M.; Bolognesi, P.; Bodo, E.; Mattioli, G.; Cartoni, A.; Avaldi, L. “An experimental and theoretical investigation of XPS and NEXAFS of 5-halouracils.” *Phys. Chem. Chem. Phys.* **20**, 6657–6667 (2018); Rondino, F.; Catone, D.; Mattioli, G.; Amore Bonapasta, A.; Bolognesi, P.; Casavola, A. R. ; Coreno, M.; O’Keeffe, P.; Avaldi, L. “Competition between electron-donor and electron-acceptor substituents in nitrotoluene isomers: A photoelectron spectroscopy and ab initio investigation.” *RSC Adv.* **4**, 5272–5282. (2014).

14 Silverstein, T. P. “The Proton in Biochemistry: Impacts on Bioenergetics, Biophysical Chemistry, and Bioorganic Chemistry” *Front. Mol. Biosci.* **8**, 764099 (2021).

15 Spicher, S.; Plett, C.; Pracht, P.; Hansen, A.; Grimme, S. “Automated Molecular Cluster Growing for Explicit Solvation by Efficient Force Field and Tight Binding Methods.” *J. Chem. Theory Comput.* **18**, 3174−3189 (2022).

16 Buelens, F. P.; Leonov, H.; de Groot, B. L.; Grubmüller, H. “ATP−Magnesium Coordination: Protein Structure-Based Force Field Evaluation and Corrections” *J. Chem. Theory Comput.* **17**, 1922−1930 (2021)
