# Supplementary material for: Effect of Protonation on the Molecular Structure of Adenosine 5′-Triphosphate: A Combined Theoretical and Near Edge X-ray Absorption Fine Structure Study
Source: J Phys Chem Lett. 2023 Nov 5;14(45):10173–80. doi: 10.1021/acs.jpclett.3c01666 (PMC10658619; doi:10.1021/acs.jpclett.3c01666)
Supplement: Supplementary file 2 — jz3c01666_si_002.pdf [file jz3c01666_si_002.pdf]

jz-2023-01666s.R1

Name: Peer Review Information for "Effect of Protonation on the Molecular Structure of Adenosine Triphosphate: a Combined Theoretical and Near Edge X-ray Absorption Fine Structure Study"

First/Second/Third Round of Reviewer Comments

Reviewer: 1

Comments to the Author

This is a thorough work providing some new insight into the molecular structure of gas-phase ATP for varied protonation state, identifying several stable protomers. The authors report on soft X-ray absorption spectra and interpret their results with theoretical calculations.

Some parts of the paper are either not well written or insufficient context is provided. This particularly applies when connecting to the aqueous phase. But this can be fixed, and along with considering my further comments, I support publication of this work in JPCL.

Specific comments and suggestions:

Page 2, line 3: It is unclear to me why using the terminology ‘action NEXAFS’. The authors should stick to what is commonly used, and this is soft X-ray absorption (XAS; or total-yield XAS in the present case); see, e.g., Fransson et al., DOI: 10.1021/acs.chemrev.5b00672. If the authors think this is inappropriate, they need to explain.

Page 2, line 10: “… are enclosed in a single frame …” is unclear and must be clarified. I suggest to re-write and break up that entire sentence. Also, the wording “This is precisely the kind of knowledge that is only obtained” is not very helpful in the abstract.

Page 3, line 10: “To accomplish this very important yet elusive challenge, …” This cannot be right. Nature knows how to do it.

Page 3, line 14: “Core-spectroscopy” can be misleading and should be replaced by ‘core-level spectroscopy’ throughout the entire manuscript.

Page 4, the entire page: Although the text provides some useful information, it oversimplifies the connection between gas-phase and aqueous phase properties. For example, hydrolysis of ATP and its associated biological function occur in the presence of metallic di-cations. There is no true gas-phase analogue; but the topic has been addressed recently [Franska et al., https://doi.org/10.1021/jasms.2c00071]. Furthermore, although the identification of the various (ring-type; Figure S9) gas-phase protomers is interesting, their number will be inevitably reduced in aqueous phase (due to fewer degrees of freedom), and hence are likely not that useful for modeling hydrated structures. Within this same context, I also urge the authors to at least mention that spectroscopic data of phosphorous would be very desirable. Note that experimental and theoretical aquoeus-phase valence electronic structure has been reported from phosphates, sugars and bases, and this is relevant for the whole structure / narrative of the present manuscript. Particularly, the phosphorous aspect is never addressed in the paper which I consider an important omission. At the bottom of the page, when the authors discuss computations, I suggest to include a discussion of structure calculations, not necessarily related to XAS. In the light of these comments, this page should be fully re-written. It would be also good if the authors comment on whether their experimental data provides any evidence for the closed form, indicative of one or two hydrogen bonds between phosphate and nitrogen of adenine as depicted in Figure S9.

Page 5, lines 7-23: This 6-line single sentence is difficult to understand.

Page 7, lines 8-10: “It is remarkable that two significantly different techniques used to study ATPH+ yield closely similar spectra. Note that in the previous study the protonated ATP is embedded in a solution, whereas in the present it is an isolated ion.” I disagree with the first part. If measurements are done right, the results should be the same. I wished that the authors expanded on the second issue. Wouldn’t one have expected a solvent-energy shift?

Page 7, lines 17-27: “… which can lead to some discrepancies since at higher photon energies more small fragment ions are formed that escape detection. “ I wasn’t aware of this; more details would be useful.

Page 7, lines 36-40: “This information comes too late.

Page 8, Figure 2c: This figure should compare the results from the present study, i.e., the computed curve (ATP TDDFT) and the here measured ATPH+ spectrum. Otherwise, I do not see the point of this figure.

Page 9, line 15: “We discuss here the interpretation of NEXAFS changes …” should be “We discuss here the XAS spectral changes …”

Page 9, line 38: “… the adenine block and the phosphate block.” The more common terminology would be “… the adenine unit and the phosphate unit”, and should be applied throughout the text.

Page 10, lines 16-20: “Remarkable resemblance between gas-phase and solvated (at low pH) ATP suggests then that such calculated structures could be relevant as a starting point in modelling the structure of solvated ATP.” This is most likely not true; please discuss in context of my above comment on hydration properties.

Page 10, lines 20-30: This text is poorly written. It can be shortened, just saying that aldditional C 1s XAS spectra must be analyzed to identify the respective molecular structures.

Page 10, line 46: “The difference cannot be explained just by different spectral resolution.” This is certainly not the first explanation coming to mind. Needs better explanation.

Page 11, line 35: “ (a) Experimental photoabsorption spectra …” Should say “ (a) Experimental C 1s XAS spectra …” Applies similarly to Figure 4.

Page 14, lines 36-45: “However, neither spectral nor energy differences between the protomers are large enough so that the concurrence of more protomers in the measured sample can be simply ruled out. This finding is important for further modeling of the ATPaq structure, that is ATP embedded in water network, towards better understanding of its messaging function in a living organism. ” I find the wording “… spectral nor energy differences … ” unsuitable. Regarding the second sentence, one needs to be reminded that one expects a single gas-phase structure to be relevant (see my comments above).

Minor

Page 9, line 38: Should be “electron-rich …”

Page 15, line 44: It should say “… the energy resolution of the beamline …”

Page 18, line 46: The year of Ref 18 is missing.

Reviewer: 2

Comments to the Author

This experimentally-theoretical paper aims at the interpretation of the NEXAFS spectrum of protonated ATP in the gas phase. The authors use their original experimental setup to produce the ions of interest and register their X-ray absorption. The obtained spectra are compared to the calculated transitions for the C, N, and O K-edge regions. The geometries and energies of particular protonated structures of ATP were calculated using the conformer search as implemented in the CREST program using the tight-binding GFN2-xTB Hamiltonian. The energetics of the most stable structures were then refined at the M06-2X level. The authors demonstrate that ATP protonation leads to a significant change in the inner-shell C and N photoexcitation with respect to neutral ATP. Their results suggest that protonation at the N3 site of ATP is responsible for the actual NEXAFS spectrum. However, the similarity of spectra for the N2 and N1 protonated ATP and their small energy difference do not exclude an involvement of other protomers in the measure spectroscopic characteristics. The paper is clearly written and experiments and calculations seem to present a pretty solid work.

- My major concern is related to the motivation of their studies. This is true that NEXAFS spectra have not been reported for neutral or protonated ATP. This fact justifies the publication of these results in a specialized journal. However, for a JPC Lett article, its findings should be published urgently and the results should be of interest to the general public. I do not see these requirements being fulfilled by this work.

- The reason for the studies described in the paper is not very obvious. The authors put a lengthy discussion suggesting that the comparison of the NEXAFS spectrum for gas phase adenine to that of solvated ATP (Kelly et al.) is not especially practical. Although it is true, also the subject formulated by the authors does not seem to be close to a practical situation. From the very beginning, the authors emphasize the role of ATP in living organisms. One should however remember that in the physiological environment pH is around 7. Under such conditions, ATP is not protonated. Just in opposite the phosphate residue is highly deprotonated – so in an aqueous solution at the physiological pH ATP is in the form of polyanion. Therefore, it is difficult to imagine physiological situations in which the protonated forms of ATP as considered in the evaluated work would be important. The authors should justify better the choice of the object of undertaken studies.

- The author say: “To closely investigate the structure of protonated ATP and its correspondence to the measured NEXAFS spectra, we have performed a comprehensive set of multilevel tight-binding and (time dependent) density functional theory calculations, that include the exploration of rotational isomers of ATP in gas phase, the exploration of all possible protonated isomers (protomers), DFT calculations of free energies of such four protomers after full geometry optimization and, finally, TDDFT

calculations of NEXAFS spectra (see Methods and SI for more details)" and then "For different protonation sites (see Figure 1a), the relative free energies of protomers are listed as following: Protomer 1 = +0.05 eV; Protomer 2 = +0.09 eV; Protomer 3 = 0.0 eV; Protomer 4 = + 0.53 eV". It is completely unclear at which level these relative energies were calculated. Without reading the methods section in SI one may suspect that the energies were obtained at the tight-binding level which is incomprehensible when one realizes that tight-binding is a semiempirical version of DFT and ATP is not a that large molecule. Only careful reading of the indicated section of SI suggests that they used the M06-2X functional for the refinement of energetics. The level at which energetics was obtained should, however, be clearly stated in the main text.

- The authors claim that only a slight prevalence of protomer 3 should be observed: "the calculations suggest that the three lowest-energy protomers 1-3 involving different N atoms of the adenine block are close in energy, with a slight prevalence of protomer 3". However, one can easily calculate for the reported relative free energies that at room temperature the ratio protomer 3: protomer 1 amounts to ca. 7 while protomer 3: protomer 2 to ca. 33. Thus the equilibrium concentration of protomer 3 is 7 folds larger than that of protomer 1 and 33 folds larger than that of protomer 2. I would not say that "the prevalence is slight". The equilibrium mixture of monoprotonated ATP should be dominated by protomer 3 and accordingly, the observed NEXAFS spectrum should be almost completely reproduced by the spectrum calculated for protomer 3, which seems to be the case. Indeed, the authors notice: "A closer analysis of all the results suggests that protonation of N3, rather than N1 or N2, yields calculated spectra in slightly better agreement with measurement…"

Author's Response to Peer Review Comments:

Effect of protonation on the molecular structure of adenosine triphosphate: a combined theoretical and action NEXAFS study

Giuseppe Mattioli, Robin Schürmann, Chiara Nicolafrancesco, Alexandre Giuliani, and Aleksandar R. Milosavljević

Reply to referees' report

(report sent to authors on 17-Jul-2023)

Dear Editor,

We would like to thank the Reviewers for their time spent to read and review our work, as well as for the detailed reports that can lead to an improved manuscript, suitable for publication in JPCL.

We hope that after these revisions the present paper shoes the urgency and warrants the publication in JPCL.

Reply to referees' comments and questions: Reviewer: 1

Recommendation: This paper may be publishable, but major revision is needed; I

would like to be invited to review any future revision.

Comments:
This is a thorough work providing some new insight into the molecular structure of gas-phase ATP for varied protonation state, identifying several stable protomers. The authors report on soft X-ray absorption spectra and interpret their results with theoretical calculations.

Some parts of the paper are either not well written or insufficient context is provided. This particularly applies when connecting to the aqueous phase. But this can be fixed, and along with considering my further comments, I support publication of this work in JPCL.

We are deeply grateful to the Reviewer for His/Her support and suggestions aimed at improving the manuscript. We have implemented the suggestions and corrections in the revised version.

Specific comments and suggestions:

Comment 1: Page 2, line 3: It is unclear to me why using the terminology 'action NEXAFS'. The authors should stick to what is commonly used, and this is soft Xray absorption (XAS; or total-yield XAS in the present case); see, e.g., Fransson et al., DOI: 10.1021/acs.chemrev.5b00672. If the authors think this is inappropriate, they need to explain.

Reply to Comment 1: We agree with the Reviewer that the terminology "action NEXAFS" might not be the most convenient. We have originally used this terminology in many of our pioneering papers related to the present experimental technique to distinguish our technique from standard XAS or NEXAFS experiments, where typically a total electron or ion yield is measured as a function of photon energy. In the present case, however, we record a highresolution mass spectrum at each photon energy step (and for each irradiation event at that energy), therefore performing a mass spectrometry at each step. The NEXAFS spectrum is then only one information extracted from this multidimensional matrix by simply extracting normalized integrated mass spectra (but within the used m/z limits, as explained below) as a function of the photon energy. Indeed, the terminology "action NEXAFS" might not be clear to all readers and might be confused with other techniques as it is used widely. We could use the terminology suggested by the Reviewer: soft X-ray absorption (XAS), which is in principle true. But this might be too broad as well. We therefore propose to simply keep the standard terminology Near-edge x-ray absorption fine structure (NEXAFS, avoiding "action") when referring to the measured absorption spectra, and to use terminology Near edge x-ray absorption mass spectrometry (NEXAMS) when referring to our experimental technique.

The wording "action NEXAFS" has been removed from the abstract and from the manuscript text.

Comment 2: Page 2, line 10: "… are enclosed in a single frame …" is unclear and must be clarified. I suggest to re-write and break up that entire sentence. Also, the wording "This is precisely the kind of knowledge that is only obtained" is not very helpful in the abstract.

Reply to Comment 2: We accept this criticism. The sentence has been changed in the revised abstract.

Comment 3: Page 3, line 10: "To accomplish this very important yet elusive challenge, …" This cannot be right. Nature knows how to do it.

Reply to Comment 3: We accept this criticism, as well. We have modified this part to be less literal and more rigorous.

Comment 4: Page 3, line 14: “Core-spectroscopy” can be misleading and should be replaced by ‘core-level spectroscopy’ throughout the entire manuscript.

Reply to Comment 4: We accept this suggestion and we have modified this expression throughout the whole text.

Page 4, the entire page:

Comment 5: Although the text provides some useful information, it oversimplifies the connection between gas-phase and aqueous phase properties. For example, hydrolysis of ATP and its associated biological function occur in the presence of metallic di-cations. There is no true gas-phase analogue; but the topic has been addressed recently [Franska et al., https://doi.org/10.1021/jasms.2c00071].

Reply to Comment 5: We thank the Reviewer for His/Her criticism that led to the improvement of the manuscript regarding its relevance for real biological systems. Part of the reply to this comment is also connected to the main criticism of the Reviewer 2 (below) and stimulated a substantial revision of the abstract and the introduction to clarify the general importance and urgency of the present study. We are also grateful for the useful reference, which has been cited and commented in the revised manuscript.

Comment 6: Furthermore, although the identification of the various (ring-type; Figure S9) gas-phase protomers is interesting, their number will be inevitably reduced in aqueous phase (due to fewer degrees of freedom), and hence are likely not that useful for modeling hydrated structures.

Reply to Comment 6: We agree with the Reviewer that the present theoretical study of various gas-phase protomers cannot be automatically or fully translated to the aqueous phase. Still, previous studies implied that protonation patterns in solution should be at least partly preserved during and after transfer into the gas phase [Nucleic Acids Research, 2019, Vol. 47, No. 14 7223–7234]. Therefore, our experimental study of the isolated ESI ions, and a comprehensive theoretical modeling of such systems, should be relevant, at least as a starting point. Additional theoretical modeling of hydrated systems would allow a more profound study, but an extensive study would be very far from the scope of the present manuscript. Nevertheless, we have been stimulated by this comment and we have added to the Supporting information a new section S6 reporting preliminary results on the interaction of water molecules with ATP and $ATPH^+$ in hydrated and fully solvated structures. Our new results suggest indeed that folded or coiled structures are preserved in gas phase clusters containing a few water molecules and are stabilized rather then disrupted even in water solution. On the grounds of such additional results, we believe that our suggestion of a seminal role of structures investigated in gas phase as starting models for further studies in complex environments is now less speculative and more interesting for the

readership.

We thank again the Reviewer for His/Her stimulating criticism; We have implemented the considerations expressed above in the revised manuscript and
SI.

Comment 7: Within this same context, I also urge the authors to at least mention that spectroscopic data of phosphorous would be very desirable. Note that experimental and theoretical aquoeus-phase valence electronic structure has been reported from phosphates, sugars and bases, and this is relevant for the whole structure / narrative of the present manuscript. Particularly, the phosphorous aspect is never addressed in the paper which I consider an important omission.

Reply to Comment 7: We agree with the Reviewer on the fact that NEXAFS curves at the P2p edge would represent an interesting complement of our data. However we had good reason to retain our data for subsequent disclosure. Apart from the NEXAFS curves, our data also allow to study selected fragment intensity yields. We found that different fragmentation channels had significantly different intensities when the photon energy was loaded into the phosphorous part of ATP and we are working on this to produce a report. Moreover, the format and the length limit of JPCL do not allow to extend our manuscript that much. Also, that would be out of the scope of the manuscript, considering that in the present case we have investigated the relationship between the structure of ATP and its protomers and their electronic properties as probed by experimental/theoretical core spectroscopies at the C, N and O edges. Finally, the calculation of NEXAFS at L edges cannot be currently performed using the same TDDFT framework used for K edges, due to theoretical complications in the treatment of the spin-orbit interaction. This would force us to introduce and assess a different calculation method and we prefer to refrain from introducing further complexity in this letter.

Comment 8: At the bottom of the page, when the authors discuss computations, I suggest to include a discussion of structure calculations, not necessarily related to XAS.

Reply to Comment 7: In the revised manuscript, we have inserted a few changes aimed at making the reader more aware of theoretical methods, specially related to the level of theory used to calculate free-energy estimates, and at isolating the main results of our structural analysis, i.e., the formation of similar coiled/folded stable structures pinned by H bonds in all cases. We think that in the economy of this letter this information is crucial to introduce the comparison between calculated and measured NEXAFS spectra as an essential link to establish structure-property relationships. We have limited the discussion of structural results to this short description due to the strict limits imposed by the JPCL format, but we have enriched the discussion on the structural properties of the investigated molecules in different conditions by adding a new section in the SI.

Comment 9: In the light of these comments, this page should be fully re-written.

Reply to Comment 9: We have almost entirely re-written page 4, as suggested by the reviewer.

Comment 10: It would be also good if the authors comment on whether their experimental data provides any evidence for the closed form, indicative of one or two hydrogen bonds between phosphate and nitrogen of adenine as depicted in Figure S9.

Reply to comment 10: the formation of H-bonds such as P=O...H-N ones surely acts as a modifier of the local environment of core electrons which, in turn, is going to affect the adsorption/photoemission processes involving such core levels. We have recently discussed such modifications in the case of XPS spectra of uracil clusters in gas phase (Mattioli et al., Sci. Rep. **2020**, 10, 1; Phys. Chem. Chem. Phys. **2021**, 23, 15049), pure and mixed with water molecules, where the intramolecular connectivity is provided by a dense network of C=O...H-N H-bonds in the case of pure uracil. Based on this analysis, we

can expect that the energy level of N or O core holes involved in such bonds can be shifted by an amount that can reach 1 eV. However, NEXAFS spectra of ATP, even the simplest N 1s, are much more crowded than XPS spectra of uracil, so that a systematic assignment of the effect of H-bonds on the spectra cannot be provided but in very evident cases. The most prominent one is represented by N4, which is discussed at a very detailed level in the “Fine analysis of NEXAFS simulations” section S3 of the Supporting information. We recall here briefly what is discussed there: the measured N 1s NEXAFS spectrum of adenine shows a wide gap between the first, sharp, and the second, broader, feature of the N spectrum. This gap is narrower in the case of ATP (and also $ATPH^+$). Theoretical NEXAFS calculations, which closely reproduce adenine and ATP spectra, clearly indicate that the red shift of N4 in ATP (around 0.7 eV) is responsible for such narrowing, and that the shift is caused by the formation of a P=O...H-N H-bond, shown in Figure S9, with the electron rich P=O group that provides a screening of the core hole through the H-bond which is compatible with the red shift. This narrowing can be therefore considered as an indirect yet sound experimental evidence of the formation of a coiled structure of ATP in gas phase, pinned by H-bonds. No complementary evidence can be extracted from the O 1s NEXAFS spectra of ATP or $ATPH^+$, because they are a convolution of a very large number of weak transitions where it is impossible to assign a single component.

Comment 11: Page 5, lines 7-23: This 6-line single sentence is difficult to understand.

Reply to comment 11: We have revised this sentence to be more readable. We thank the Reviewer for His/Her suggestion.

Comment 12: Page 7, lines 8-10: “It is remarkable that two significantly different techniques used to study ATPH+ yield closely similar spectra. Note that in the previous study the protonated ATP is embedded in a solution, whereas in the present it is an isolated ion.” I disagree with the first part. If measurements are done right, the results should be the same. I wished that the authors expanded on the second issue. Wouldn’t one have expected a solvent-energy shift?

Reply to Comment 12: We agree with the Reviewer that the excitement about the two measurements showing the same (as they should) is a bit overexpressed and thus misleading with respect to what is important – possible influence of the solvent. This part is revised in the new version. We give below several additional comments:

- There are limitations to discuss a possible solvent-energy shift according to the experimental data. Kelly et all do not report the accuracy (or the calibration procedure) of their photon energy scale. Moreover, even for the adenine experimental data, the authors do not report the experimental uncertainty of their photon energy scale. So, unfortunately, we cannot accurately discuss if there is an energy shift induced by solvation as revealed by the experimental data.
- On the other hand, by presuming that the position of the ATPaq is well calibrated (note that we have received the data from the authors through a personal communication), a difference of only about 0.1 eV is smaller than our stated uncertainty of the photon energy scale. Therefore, we can only say that a possible solvent-energy shift is below measurable uncertainty, which means that the presence of solvent molecules does not significantly influence C, N core-transition energies (note that this is only if we presume that the published data are with similar or smaller uncertainty).
- Moreover, the shape of the two spectra is very similar (see also the reply to the next Comment below), suggesting that the possible presence of solvent in the sample does not affect significantly the relative position of the resonances in $ATPH^+$.

- Finally, on the side of simulations we have added the new Section S6 in the SI, where we provide a preliminary discussion, stimulated by both Reviewer's comments, of the transition between gas phase, solvent-free ATP ($ATPH^+$) and solvated ATP ($ATPH^+$), through gas phase clusters where one ATP ($ATPH^+$) molecule is surrounded by a small amount of water molecules. Regarding possible solvent shifts of NEXAFS spectra, we note that the formation of small clusters in gas phase containing up to ten water molecules in direct contact with ATP or $ATPH^+$ is generally accompanied by small shifts, compatible with the uncertainty on the experimental energy scale of photons and negligibly affecting the shape of the spectra. Remarkably, only the addition of 10 water molecules to neutral ATP suggests the formation of a neutral stable structure in which one of the phosphate units is deprotonated and screened by water and the excess proton is transferred to N2 (and not to N3, which is the lowestenergy site in gas-phase for $ATPH^+$ cations). This would induce strong changes in the spectrum, as now shown in the SI, and it is, therefore, ruled out by comparison with the two ATP spectra measured using pH=7.5 and pH=2.5 solutions.

Such considerations have been summarized in the revised manuscript and addressed in more detail in the SI.

Comment 13: Page 7, lines 17-27: "… which can lead to some discrepancies since at higher photon energies more small fragment ions are formed that escape detection. " I wasn't aware of this; more details would be useful.

Reply to Comment 13: Generally, with increasing the photon energy deposited in a biomolecule, the fragmentation pattern shifts towards small fragments. For example, we reported previously for SubP peptide that the yield of small singly charged backbone fragments increased markedly with increasing the photon energy than that of larger doubly and triply charged ions, arguing that large doubly and triply charged backbone fragments apparently cannot survive higher energy load into the peptide system. A similar effect had been reported previously in a study of near C-edge X-ray absorption mass spectrometry of a smaller protonated leucine encephalin [J. Phys. Chem. A, 2012, 116, 10745– 10751].

On the other hand, what we record for each photon energy step is a mass spectrum and the low mass/charge limit (as well as high m/z limit) depends on the selected m/z range for the LTQ ion trap mass spectrometer. In the present case, the lower m/z limit was m/z 115. Therefore, if there was an increased yield of fragments smaller than m/z 115 at higher photon energies, that would not be detected, since our "total ion yield" is defined by the range of the mass spectrum. That could therefore produce a different relative shape of the NEXAFS curve with respect to the one where a total electron yield is measured (moreover, in the case of the electron yield, the multiple electron ejection above the core-IP would produce an increased sensitivity per an ionisation event, so the opposite effect).

Comment 14: Page 7, lines 36-40: "This information comes too late.

Reply to Comment 14: In the revised manuscript this sentence (slightly modified) has been moved above and it is now at the beginning of the page in the first paragraph.

Comment 15: Page 8, Figure 2c: This figure should compare the results from the present study, i.e., the computed curve (ATP TDDFT) and the here measured ATPH+ spectrum. Otherwise, I do not see the point of this figure.

Reply to Comment 15: We understand that the presentation of all results (present experimental/theoretical, previous experimental for ATP from solution at different pH and previous gas-phase adenine) is complex. We have also spent quite some time discussing the best way to do it, to be informative, simple and within the short JPCL page limits. We will try to explain our choice below, replying at the same time to the Reviewer's comment:

- The comparison between the results from the present study, i.e., the computed curves of ATPH+ (TDDFT) and the measured ATPH+ spectrum is already presented in Fig. 2d. However, there are 3 computed curves for ATPH+ (for the 3 different investigated protomers).
- On the other hand, we consider inappropriate to present a comparison between the computed curve for ATP (TDDFT, which is now only one curve, there are no protomers) and the present experiment because we could not measure neutral ATP isolated in the gas phase.
- Still, the theoretical results (including the computed NEXFAS) are as much the results of the present study as the experimental ones. These are highlevel unprecedented calculations for such a complex system, and a comprehensive theoretical work. Therefore, we wanted to present the calculated curves in the main article for the systems that were not measured in our experiment, that is ATP and adenine, and compare them with available experimental results. Such presentation, in a single Figure, shows how the shape of the NEXAFS spectrum is changing from adenine to neutral ATP and then from the latter to protonated ATP. At the same time, it shows the reliability of our theoretical setup based on the practically perfect agreement between the present calculations and previously published experimental results for adenine and neutral ATP (that is ATPaq at neutral pH).
- Therefore, we have chosen a presentation that consists of four frames in one figure. The first frame (a) compares all experimental results (for all discussed systems). The frames (b) and (c) compare the present theoretical results with the existing published exp curves (and show the transition of the spectrum), and finally the frame (d) compares present theoretical results with the present measurements.

Considering all these points, we are inclined to leave the figures as they are in the revised manuscript, following the logic explained above and accordance with the text in the manuscript.

Comment 16: Page 9, line 15: "We discuss here the interpretation of NEXAFS changes …" should be "We discuss here the XAS spectral changes …"

Reply to Comment 16: Please, see the reply to Comment 1.

Comment 17: Page 9, line 38: "… the adenine block and the phosphate block." The more common terminology would be "… the adenine unit and the phosphate unit", and should be applied throughout the text.

Reply to Comment 17: This has been changed in the revised version.

Comment 18: Page 10, lines 16-20: "Remarkable resemblance between gasphase and solvated (at low pH) ATP suggests then that such calculated structures could be relevant as a starting point in modelling the structure of solvated ATP." This is most likely not true; please discuss in context of my above comment on hydration properties.

Reply to Comment 18: We agree with the Reviewer on the fact that our pristine sentence was a bit too enthusiastic and not accompanied by sound experimental or theoretical evidence. Stimulated by His/Her criticism, we have performed further calculations, as already discussed in detail in our reply to the Reviewer's
Comment 6. In the revised manuscript, we have therefore rephrased the sentence in a more cautious way, without completely withdrawing our suggestion.

Comment 19: Page 10, lines 20-30: This text is poorly written. It can be shortened, just saying that aldditional C 1s XAS spectra must be analyzed to identify the respective molecular structures.

Reply to Comment 19: We thank the Reviewer for this criticism. This part of the text has been revised and shortened in agreement with His/Her suggestion.

Comment 20: Page 10, line 46: "The difference cannot be explained just by different spectral resolution." This is certainly not the first explanation coming to mind. Needs better explanation.

Reply to comment 20: We agree that this sentence is not well formulated. This part is commenting on the similarities between XAS spectra of neutral and protonated molecules. We have removed the confusing sentences and reformulated this part.

Comment 21: Page 11, line 35: " (a) Experimental photoabsorption spectra …" Should say " (a) Experimental C 1s XAS spectra …" Applies similarly to Figure 4.

Reply to Comment 21: This has been modified accordingly.

Comment 22: Page 14, lines 36-45: "However, neither spectral nor energy differences between the protomers are large enough so that the concurrence of more protomers in the measured sample can be simply ruled out. This finding is important for further modeling of the ATPaq structure, that is ATP embedded in water network, towards better understanding of its messaging function in a living organism. " I find the wording "… spectral nor energy differences … " unsuitable. Regarding the second sentence, one needs to be reminded that one expects a single gas-phase structure to be relevant (see my comments above).

I find the wording "… spectral nor energy differences … " unsuitable

Reply to Comment 22a: This has been changed in the revised version. We thank the Reviewer for His/Her suggestion.

Regarding the second sentence, one needs to be reminded that one expects a single gas-phase structure to be relevant (see my comments above).

Reply to Comment 22b: We believe that the Reviewer is referring here to His/Her comment that the number of gas-phase protomers will be inevitably reduced in aqueous phase due to fewer degrees of freedom, and hence they are likely not that useful for modeling hydrated structures. We replied and discussed this suggestion above, also citing a new reference. We believe that the modeling of isolated $ATPH^+$ and the relationship between investigated structures and calculated/measured NEXAFS spectra are not irrelevant for further investigation of hydrated and/or solvated ATPH. Preliminary results, readily inserted in a new section of the SI and also discussed in more detail above (see in particular the replies to the Reviewer's Comments 6 and 12), may provide a better support to the idea that different

protomers should be considered, depending on the conditions in which they are obtained and measured. A few changes have been introduced in the revised manuscript in agreement with these considerations.

Minor

Page 9, line 38: Should be “electron-rich …”
Page 15, line 44: It should say “… the energy resolution of the beamline …” Page 18, line 46: The year of Ref 18 is missing.

These points have been corrected in the revised manuscript. We thank the Reviewer for His/Her suggestions.

Reviewer: 2

Recommendation: Reconsider as an article in The Journal of Physical Chemistry A/B/C.

Comments:
This experimentally-theoretical paper aims at the interpretation of the NEXAFS spectrum of protonated ATP in the gas phase. The authors use their original experimental setup to produce the ions of interest and register their X-ray absorption. The obtained spectra are compared to the calculated transitions for the C, N, and O K-edge regions. The geometries and energies of particular protonated structures of ATP were calculated using the conformer search as implemented in the CREST program using the tight-binding GFN2-xTB Hamiltonian. The energetics of the most stable structures were then refined at the M06-2X level. The authors demonstrate that ATP protonation leads to a significant change in the inner-shell C and N photoexcitation with respect to neutral ATP. Their results suggest that protonation at the N3 site of ATP is responsible for the actual NEXAFS spectrum. However, the similarity of spectra for the N2 and N1 protonated ATP and their small energy difference do not exclude an involvement of other protomers in the measure spectroscopic characteristics. The paper is clearly written and experiments and calculations seem to present a pretty solid work.

- My major concern is related to the motivation of their studies. This is true that NEXAFS spectra have not been reported for neutral or protonated ATP. This fact justifies the publication of these results in a specialized journal. However, for a JPC Lett article, its findings should be published urgently and the results should be of interest to the general public. I do not see these requirements being fulfilled by this work.
- The reason for the studies described in the paper is not very obvious. The authors put a lengthy discussion suggesting that the comparison of the NEXAFS spectrum for gas phase adenine to that of solvated ATP (Kelly et al.) is not especially practical. Although it is true, also the subject formulated by the authors does not seem to be close to a practical situation. From the very beginning, the authors emphasize the role of ATP in living organisms. One should however remember that in the physiological environment pH is around 7. Under such conditions, ATP is not protonated. Just in opposite the phosphate residue is highly deprotonated – so in an aqueous solution at the physiological pH ATP is in the form of polyanion. Therefore, it is difficult to imagine physiological situations in which the protonated forms of ATP as considered in the evaluated work would be important. The authors should justify better the choice of the object of undertaken studies.

We thanks the Reviewer for His/Her criticism, which have been used to improve the revised manuscript. In agreement with His/Her concern, we have revised the abstract, the introduction and the conclusion to account for the criticism and to show the relevance of the present study on protonated ATP (and different protomers) for realistic systems. We have also added new references, including some suggested by the other Reviewer.

It is indeed true that ATP is highly acidic and appears under deprotonated forms in solution, as also discussed in the new section S6 of the SI. However, protonation of the adenine unit is not irrelevant to consider. Several works have reported the influence of adenine protonation in the structure of noncoding RNA [Baisden, J.T., Boyer, J.A., Zhao, B. et al. Visualizing a protonated RNA state that modulates microRNA-21 maturation. Nat Chem Biol 17, 80–88 (2021)] and mRNA [B. Houck-Loomis, M. A. Durney, C. Salguero, N. Shankar, J. M. Nagle, S. P. Goff, V. M. D'Souza, Nature 2011, 480, 561–564]. Furthermore, the interaction of protonated adenine with negatively charged phosphate has been suggested to be of importance in the structuration of the molecule [Roger Phillips, S. J., Chem. Rev. 1966, 66, 5, 501–527; P. Wang, R. M. Izatt, J. L. Oscarson, S. E. Gillespie, J. Phys. Chem. 1996, 100, 9556–9560]. Hence, the picture of a fully deprotonated ion may not always be adequate to describe ATP in solution. Moreover, the study of the protonation sites of adenine and their influence on the electronic structure is of importance not only to the present case, but also to other fundamental molecules, such as RNA, thereby reaching a broader audience [Nucleic Acids Research, 2019, Vol. 47, No. 14 7223–7234]. Indeed, these aspects were missing in the previous version of our manuscript as noticed by the Reviewer, and have been added in the revised manuscript.

Finally, we would like to note again a remarkable agreement between the experimental absorption spectra obtained for an isolated cation in gas phase and a sample obtained by jet-spray technique applied to a low-pH ATP solution. Such very similar spectroscopic signature implies that the structure of isolated, protonated ATP could be relevant to understand the structure of protonated ATP in a solution. This consideration has also stimulated further calculations, discussed in the new Section S6 of the SI, showing among obvious differences significant structural similarities between neutral and protonated ATP in different conditions (isolated in gas phase, hydrated in gas phase, fully solvated). Such results permit a stronger link between the core of our study, devoted to the joint experimental/theoretical analysis of structure-property relationships through the comparison of calculated and measured NEXAFS spectra, and more general considerations on the properties of ATP and $ATPH^+$ in more realistic conditions.

Comment 2: The author say: "To closely investigate the structure of protonated ATP and its correspondence to the measured NEXAFS spectra, we have performed a comprehensive set of multilevel tight-binding and (time dependent) density functional theory calculations, that include the exploration of rotational isomers of ATP in gas phase, the exploration of all possible protonated isomers (protomers), DFT calculations of free energies of such four protomers after full geometry optimization and, finally, TDDFT calculations of NEXAFS spectra (see Methods and SI for more details)" and then "For different protonation sites (see Figure 1a), the relative free energies of protomers are listed as following:
Protomer 1 = +0.05 eV; Protomer 2 = +0.09 eV; Protomer 3 = 0.0 eV; Protomer 4 = + 0.53 eV". It is completely unclear at which level these relative energies were calculated. Without reading the methods section in SI one may suspect that the energies were obtained at the tight-binding level which is incomprehensible when one realizes that tight-binding is a semiempirical version of DFT

and ATP is not a that large molecule. Only careful reading of the indicated section of SI suggests that they used the M06-2X functional for the refinement of energetics. The level at which energetics was obtained should, however, be clearly stated in the main text.

Reply to Comment 3: The free energies of ATP and of all protomers have been actually calculated using a compound method, which is thoroughly explained in the SI. We have decided not to provide a simplified yet incomplete information because we have not merely optimized the molecules using a given DFT exchange-correlation functional and a given basis set. Instead, we have first generated an ensemble of conformers-rotamers using tight-binding and a search algorithm, then we have refined a large subset of the low-energy sorted structures using the D3-dispersion-corrected M06-2X functional and the large def2-TZVPP basis set. Finally, we added to this DFT energy the contribution of the ZPE and of the electronic, rotational and vibrational entropy, calculated using the r^2SCAN functional and its tailored mTZVPP basis set. A clear indication of the method used to calculate the relative energies would be too long and also too technical for a broad readerhip. We have therefore modified the main text in partial agreement with the Reviewer's suggestion, by inserting in the caption of Figures 1 and S9 the indication that geometry optimizations have been performed by using the dispersion-corrected M06-2X functional and the def2TZVPP basis set, as this is not only formally correct but discloses also in the main text the main DFT level of theory used to calculate the free energies.

Comment 3: The authors claim that only a slight prevalence of protomer 3 should be observed: "the calculations suggest that the three lowest-energy protomers 13 involving different N atoms of the adenine block are close in energy, with a slight prevalence of protomer 3". However, one can easily calculate for the reported relative free energies that at room temperature the ratio protomer 3: protomer 1 amounts to ca. 7 while protomer 3: protomer 2 to ca. 33. Thus the equilibrium concentration of protomer 3 is 7 folds larger than that of protomer 1 and 33 folds larger than that of protomer 2. I would not say that "the prevalence is slight". The equilibrium mixture of monoprotonated ATP should be dominated by protomer 3 and accordingly, the observed NEXAFS spectrum should be almost completely reproduced by the spectrum calculated for protomer 3, which seems to be the case. Indeed, the authors notice: "A closer analysis of all the results suggests that protonation of N3, rather than N1 or N2, yields calculated spectra in slightly better agreement with measurement…"

Reply to Comment 2: We agree in principle with the Reviewer's considerations. However, we have been cautious in the interpretation of our results, as we generally prefer to validate theoretical predictions, which at any rate we consider very carefully obtained, through comparison with experimental results. Regarding calculated free energies, the so-called "chemical accuracy" limit in ab initio calculations is set at 0.04 eV (1 kcal/mol). Even if M06-2X is considered one of the most accurate DFT exchange-correlation functionals for total energy calculations in molecules, it is credited with a mean unsigned error of around 0.12 eV in the calculation of total energy differences (see for example J. Chem. Theory Comput. 2016, 12, 2272-2284, where the issue of calculation of redox potentials of organic molecules from total energies is addressed). This estimate is also in agreement with recent, more general considerations about the placement of DFT in an ideal ladder representing chemical accuracy (Nat. Comm. 2020, 11, 5223). As our energy differences fall inside this uncertainty range, and given the fact that no conclusive argument can be drawn from the analysis of the corresponding spectra and the comparison with measurements, we did not try to stress or to over interpret our energetics results, even if they are also in agreement with the set of DLPNO-CCSD(T) calculations provided as supporting information. Finally, we also note that small perturbations (the presence of a single water molecule, see for example our

recent contribution Carlini, et al., J. Phys. Chem. B 2022, 126, 2968 on the cyclization of dipeptides) can induce the rearrangement of excess protons altering the expected distribution based on the free energies of isolated molecules. Given these considerations, as well as the fact that we have indicated a reasonable pre-eminence of protomer 3 in the measured sample, we would avoid a stronger statement.

jz-2023-01666s.R2

Name: Peer Review Information for "Effect of Protonation on the Molecular Structure of Adenosine Triphosphate: a Combined Theoretical and Near Edge X-ray Absorption Fine Structure Study"

Second Round of Reviewer Comments

Reviewer: 1

Comments to the Author

The authors have improved their manuscript but I suggest to make further improvements. Specifically, for better understanding, narrative, and readability several of the authors' replies to Reviewer #1 would be important to be actually included in the main manuscript; lengths limitation should not be a reason to not doing so. This refers particularly to parts of replies to Comments 6, 7, 10, 13, 15 as detailed next:

Comment 6: "Still, previous studies implied that protonation patterns in solution should be at least partly preserved during and after transfer into the gas phase [Nucleic Acids Research, 2019, Vol. 47, No. 14 7223–7234]. Therefore, our experimental study of the isolated ESI ions, and a comprehensive theoretical modeling of such systems, should be relevant, at least as a starting point." AND "Our new results suggest indeed that folded or coiled structures are preserved in gas phase clusters containing a few water molecules and are stabilized rather then disrupted even in water solution."

Comment 7: The authors should at least mention the potential importance of complementary measurements of phosphorous.

Comment 10: I do not find my question sufficiently answered / addressed in the main manuscript. There should be a clear discussion on whether (and how) the experiment supports the ring structure. In that very context the authors should also mention that a ring structure in aqueous solution would inevitably involve some bridging water molecule. This again leads back to the question to what extent the

comparison of gas- and aqueous-phase results is relevant. It is a central aspect of this work and must be well addressed.

Comment 13: A 1-sentence explanation should be provided.

Comment 15: "We will try to explain our choice below …" This should be done in the manuscript as well.

Other comments:

Page 3, line 19: I suggest to replace "Therefore, the present work also makes a foundation for further …" by "Therefore, the present work should be relevant for further …"

Page 5, line 25: The authors should briefly explain the different results for AMP and ATP.

Page 5, line 55: " … narrowing the gap … " does not seem to be the right wording.

Page 6, line 8: What does "bend structure" refer to? Is Ref 18 really the proper reference?

Page 6, lines 11-15: "The previous studies also implied that the protonation patterns in solution should be at least partly preserved during and after transfer into the gas phase22." This is a very vague statement, and further explanation is needed.

Author's Response to Peer Review Comments:

We are ready to accept your submission after the following non-scientific changes:

1) Highlighting: Please remove the colored text or highlights showing the changes made to the manuscript and Supporting Information files. Please upload "clean" copies for publication. You may upload annotated files separately as "Supporting Information for Review Only" files.

Done

2) Title: In both the main manuscript file and the Supporting Information, set the title in title case, with the first letter of each principal word capitalized.

Done

3) Graphics: One or more of your figures and tables includes a reference citation. Please confirm that this pertains only to data and not the figure itself. If it pertains to the use of a published image, permissions must be secured for any graphics NOT originally published by ACS or for Open Access content which permits reuse with credit only. Permission is needed if you are using another publisher's or copyright owner's figures/tables verbatim, adapting/modifying them, or using them in part. If the images are from an Open Access publisher that does not require

permission for reuse, please confirm.

Numerical data have been received as private communications from the Authors of the cited papers, and have been used to draw the original figures of the present manuscript. The communications were acknowledged in the appropriate section of the manuscript, but now they are also stated in a note in reference related to the captions of the corresponding Figures.

Reviewer(s)' Comments, if any, to Author:

Reviewer: 1

Recommendation: This paper may be publishable, but major revision is needed; I would like to be invited to review any future revision.

Comments:
The authors have improved their manuscript but I suggest to make further improvements. Specifically, for better understanding, narrative, and readability several of the authors' replies to Reviewer #1 would be important to be actually included in the main manuscript; lengths limitation should not be a reason to not doing so. This refers particularly to parts of replies to Comments 6, 7, 10, 13, 15 as detailed next:

Reply: We thank again the Reviewer for His/Her careful evaluation of our manuscript, aimed at improving the quality of our work. We have tried to satisfy all His/Her criticisms and suggestions, as detailed in the following.

Comment 6: "Still, previous studies implied that protonation patterns in solution should be at least partly preserved during and after transfer into the gas phase [Nucleic Acids Research, 2019, Vol. 47, No. 14 7223–7234]. Therefore, our experimental study of the isolated ESI ions, and a comprehensive theoretical modeling of such systems, should be relevant, at least as a starting point." AND "Our new results suggest indeed that folded or coiled structures are preserved in gas phase clusters containing a few water molecules and are stabilized rather then disrupted even in water solution."

Reply: We have included the concepts pointed out by the Reviewer in two different parts of the revised manuscript. We have also indicated more clearly that such concepts are discussed more thoroughly in Section S6.

Comment 7: The authors should at least mention the potential importance of complementary measurements of phosphorous.

Reply: We have added a sentence at the end of page 14 of the revised manuscript where we state that we don't show here NEXAFS results measured at the P L-edge, which are of potential interest for a subsequent study of different fragmentation channels following excitation, because they are of no help in the present interpretation of structure-properties relationships. A further sentence has been added in section S2 (Detailed theoretical methods) to stress the differences between K and L- edges theoretical treatment and explain why our phosphorus results require a separate publication.

Comment 10: I do not find my question sufficiently answered/addressed in the main manuscript. There should be a clear discussion on whether (and how) the experiment supports the ring structure. In that very context the authors should also mention that a ring structure in aqueous solution would inevitably involve some bridging water molecule. This again leads back to the question to what extent the comparison of gas- and aqueous-phase results is relevant. It is a central aspect of this work and must be well addressed.

Reply: We partially agree with the Reviewer. We think that the central point of our manuscript is that an extensive comparison between present and previous experimental results and a new and unprecedented, as far as we know, series of semiempirical and ab initio simulations of several molecules is able to establish sound structure-properties relationships related to isolated ATP and its protonated tautomers. In doing this, and also thanks to the stimulating comments of the reviewers, we have also explored structural and spectroscopic effects of the addition of water molecules in two separate regimes. In the former case of a few water molecules, an amount compatible with samples obtained using microjet techniques from liquid solutions, we have shown that in both neutral and protonated molecules this addition minimally alter the preferred coiled structure but for the insertion of a single water molecule as a bridge between adenine and phosphate. However, such insertion barely affects NEXAFS spectra, thus explaining the close similarity between ESI and microjet measurements and strengthening our overall comparison between theoretical and experimental results. In the latter case of a water solution, our preliminary results suggest again that molecular structures are not altered by solvation, even if phosphate is stabilized in this case by the release of two protons in solution. The simulations of ATP and $ATPH^+$ in solution, however, represent an interesting but not crucial part of the present study. Therefore, we believe that such results, albeit interesting and useful to broaden the scope of our study, should not be particularly emphasized.

We believe that the more clear references to section S6 in the main text and the new sentence added in the conclusion of the main text ("Our theoretical results also suggest that the most stable folded or coiled structures found in the case of isolated molecules are preserved in gas phase clusters containing a few water molecules and are stabilized rather then disrupted even in water solution") sufficiently address the Reviewer's criticism.

Comment 13: A 1-sentence explanation should be provided.

Reply: We agree with the Reviewer. We have added the following sentence in the experimental section of the main article: "The MS^2 spectra were measured between the low mass cutoff at m/z 115 and the precursor mass. Hence, some of the small charged fragments whose mass to charge

ration was below 115 may have escaped detection, which may potentially affects the total ion yield".

Comment 15: "We will try to explain our choice below …" This should be done in the manuscript as well.

Reply: We have added the following note in reference to the captions of figures 2 and 3, which summarizes our considerations.

"Numerical data of the measurements reported in References 10 and 14 have been received as private communications from the Authors of the publications. Among several possibilities, we have chosen to group together present and previous experimental measurements in panel (a), to favor the comparison between different molecules. In order to enhance the results of simulations, which represent an unified description of all the investigated molecules, we have separately compared measurements with calculations of akin systems in panels b-d, and we have thoroughly and extensively discussed and compared the calculations of different systems in the SI (Section S3)."

Other comments:

Page 3, line 19: I suggest to replace "Therefore, the present work also makes a foundation for further …" by "Therefore, the present work should be relevant for further …"

Reply: We thank the Reviewer for His/Her suggestion.

Page 5, line 25: The authors should briefly explain the different results for AMP and ATP.

Reply: In our opinion, this would be appropriated if we presented at least theoretical results for AMP, but this fall beyond the scope of our contribution. Shimada et al (ref XXX of the revised manuscript) ascribe the differences in relative intensity of the two main ATP and AMP peaks to protonation/deprotonation of the adenine unit, depending on pH of the starting solution. This findings are in agreement with our results, which have been obtained through close comparison between measurements and simulations. Unfortunately, the Authors measured microjet samples of protonated ATP and of neutral AMP, while comparison between protonated or between neutral ATP and AMP species is provided only in the case of solid-state samples, which are of insufficient help in our discussion on the structural properties of isolated molecules. Kelly et al, on the other hand, have studied ATP only, with a similar microjet technique, but they managed to measure both neutral and protonated ATP. Hence we used and commented their results in comparison with ours.

Page 5, line 55: " … narrowing the gap … " does not seem to be the right wording.

Reply: We have changed the sentence that is now: "including and comparing in a same study results arising from gas-phase and solvated ATP"

Page 6, line 8: What does "bend structure" refer to? Is Ref 18 really the proper reference?

Reply: Protonation of adenine leads to stronger interaction between the phosphate moiety with the adenine, which bends the structure over the ribose group. We agree that the wording is not crystal clear and we have reformulated that sentence as follow: "…, thereby leading to a sort of salt bridged structure." We are using the expression "sort of salt bridge", because salt bridges are usually described in protein structure. References 18 and 19 are adequate, since they postulate these kind of geometry for adenine in solution through thermodynamic measurements.

Page 6, lines 11-15: “The previous studies also implied that the protonation patterns in solution should be at least partly preserved during and after transfer into the gas phase 22.” This is a very vague statement, and further explanation is needed.

Reply: We agree that this sentence is not adequately formulated and not enough explicit. We have rewritten it as follow: “Previous studies have reported that different protonation patterns produced in solution are, at least partially, preserved in the gas phase upon electrospray ionization. [22] Hence a correspondence may be established between gas and solution phase studies.”
